# Conditioned media derived from HepG2 cells treated with phosphoeleganin synthetic-derived compound PE/2 improve cytokine balance and metabolic response in 3T3-L1 adipocytes

**DOI:** 10.3389/fphar.2026.1909848

**Published:** 2026-10-23

**Authors:** Federica Mormone, Giusy Ferraro, Cristina Morelli, Francesca Fiory, Serena Cabaro, Giuseppe Perruolo, Marcello Casertano, Marialuisa Menna, Pietro Formisano, Concetta Imperatore, Francesco Oriente

**Affiliations:** 1 Department of Translational Medicine, University of Naples Federico II, Naples, Italy; 2 Department of Pharmacy, University of Naples Federico II, Naples, Italy

**Keywords:** 3T3-L1 cells, conditioned media, cytokines, insulin signaling, marine-based compounds, phosphoeleganin

## Abstract

**Background:**

The liver–adipose axis regulates insulin sensitivity and lipid metabolism, and its dysregulation contributes to metabolic disorders. PE/2, a semisynthetic derivative of the marine natural product phosphoeleganin, reduces IL-6 expression and secretion, enhancing insulin sensitivity in liver cells (HepG2). However, its role in liver–adipose communication is unclear.

**Methods:**

To clarify the impact of PE/2 on liver–adipose tissue crosstalk, the conditioned media approach was used. Protein and mRNA levels were analyzed by Western blot and Real-Time RT-PCR, respectively. Lipid and glucose metabolism were evaluated by Oil Red O staining and glucose consumption assays, respectively.

**Results:**

Our results indicate that in 3T3-L1 adipocytes, conditioned media derived from PE/2-treated HepG2 cells (CM_PE/2_) increased *adiponectin* expression by 1.85-fold and reduced *interleukin-8* and *interleukin-1β* mRNA levels by 43% and 52%, respectively, compared with conditioned media alone (CM). The protective adipokine phenotype induced by CM_PE/2_ was accompanied by 1.75-fold lipid accumulation and increased expression of adipogenic markers, including *PPARγ*, *C/EBPα*, *GLUT4*, and *FABP4*. To examine whether this profile translates into enhanced insulin sensitivity, we evaluated the insulin pathway. Interestingly, CM from insulin-stimulated HepG2 cells (CM_Ins_) did not significantly increase insulin receptor (INSR) or Akt phosphorylation compared with CM from 3T3-L1 adipocytes. However, CM derived from HepG2 co-treated with insulin and PE/2 (CM_Ins+PE/2_) increased INSR and Akt phosphorylation by 2.13- and 1.63-fold, respectively, compared to CM, and by 1.83- and 1.42-fold, respectively, compared to CM_Ins_. Consistently, 3T3-L1 glucose consumption significantly improved in the presence of CM_Ins+PE/2_. Treatment with the PI3K/Akt inhibitor LY294002 reversed the effects of CM_Ins+PE/2_. Finally, supplementing recombinant IL-6 into CM_PE/2-_treated cells reduced the effect of this compound on the expression of inflammatory and adipogenic genes and on glucose consumption in 3T3-L1 adipocytes, corroborating the hypothesis that IL-6 is involved in the action mediated by PE/2.

**Conclusion:**

These findings suggest that PE/2 improves the metabolic–inflammatory profile in adipocytes by modulating hepatocyte–adipocyte crosstalk, highlighting its implication for metabolic dysfunctions associated with impaired liver-adipose communication.

## 1 Introduction

The liver and adipose tissue (AT) are two primary metabolic organs that provide energy to the entire organism by storing and regulating the availability of systemic nutrients. However, emerging evidence suggests that these organs can also be considered active endocrine players, which, by secreting cytokines, hepatokines, and adipokines, convey metabolic signals throughout the body via the circulatory system. Thus, inter-organ communication between the liver and AT is crucial for maintaining systemic homeostasis and optimizing energy utilization (Ye et al., 2017; Zhang et al., 2023; Tattoli et al., 2024). Dysregulation of the liver-adipose tissue metabolic axis can impair energy balance and contribute to various pathological conditions, including insulin resistance, inflammation, metabolic dysfunction-associated fatty liver disease (MAFLD), and obesity. Considering that current drugs are not entirely free of side effects, the discovery of new chemical entities that enhance insulin sensitivity is crucial for developing novel medicines to prevent and treat metabolic diseases.

Marine organisms produce secondary metabolites that are structurally distinct from those produced by terrestrial ones, due to the unique biosynthetic milieu (high salinity, pressure, and temperature). Unusual functional groups such as isocyanates, isonitriles, phosphates, and sulfates, as well as halogenated functionalities, are predominantly found in marine metabolites. This represents a rich source of chemodiversity to which a high potential for discovering novel therapeutics with unexplored mechanisms of action is associated.

Among the marine natural products (MNPs), a novel phosphorylated polyketide, phosphoeleganin (PE), was isolated from the Mediterranean ascidian *Aplidium elegans*, and its complex structure and configuration were determined by extensive use of Two-Dimensional Nuclear Magnetic Resonance and microscale chemical degradation and/or derivatization (Imperatore et al., 2016; Luciano et al., 2017). PE has been shown to inhibit protein tyrosine phosphatase 1B (PTP1B), thereby improving insulin signaling in skeletal muscle cells (Genovese et al., 2021). Considering that marine resources are finite and subject to temporal variability in their availability, this result encouraged the rational design and synthesis of simplified or modified PE-semisynthetic derivatives for more in-depth Structure-Activity Relationship (SAR) studies. A set of small-sized and less complex fragments of PE was obtained by gradually disassembling the whole structure skeleton of the parent molecule. The two most potent PTP1B inhibitors in the series, the semisynthetic fragment PE/3 and the synthetic analogue 1, possessed the same regiochemistry and absolute configuration as phosphoeleganin at the 1,2-monophosphorylated diol system, suggesting that position and spatial orientation of the phosphate group are a key requirement for PTP1B inhibition (Figure 1).

**FIGURE 1:**
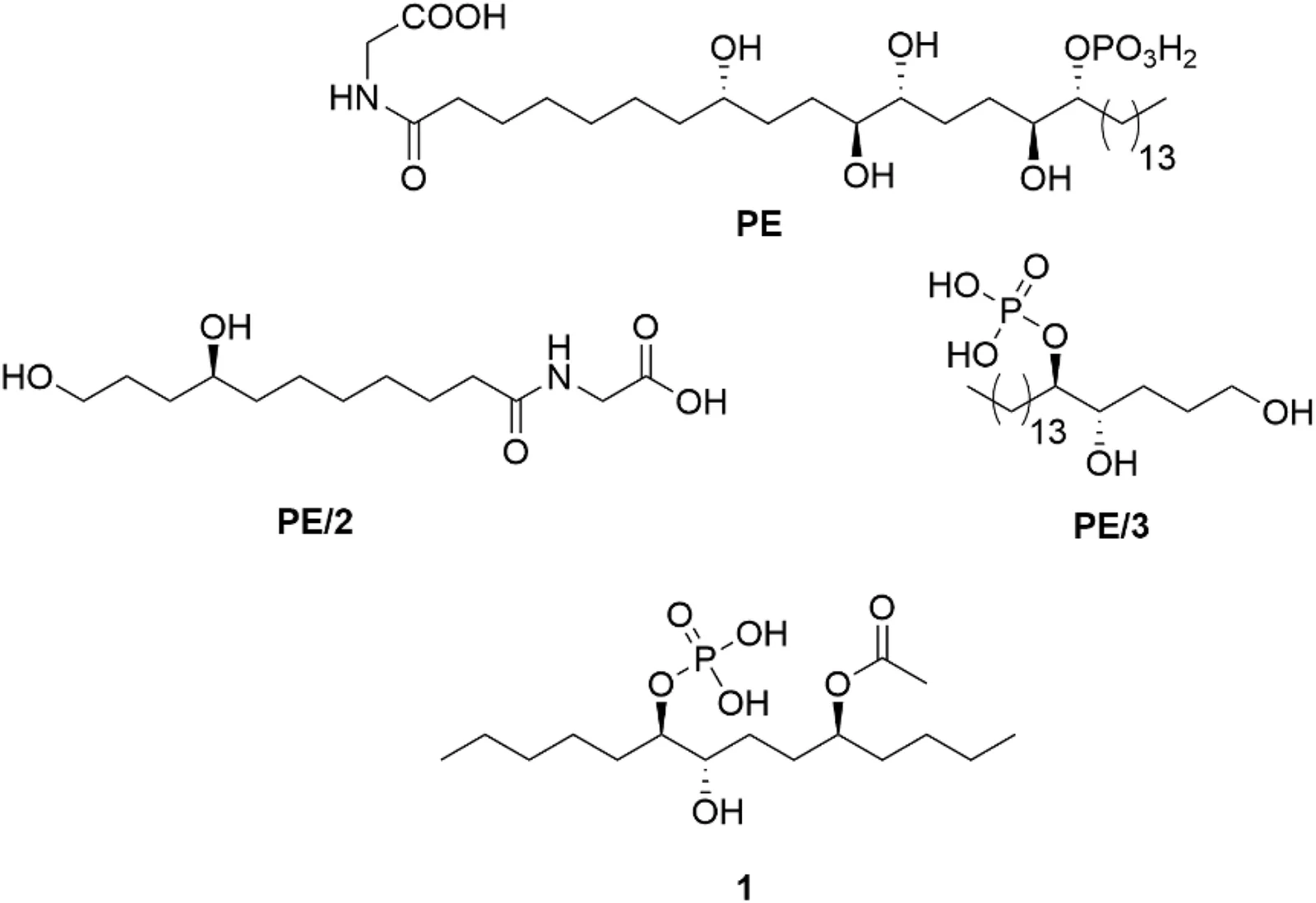
Chemical structures of PE, its semisynthetic oxidative cleavage products PE/2 and PE/3, and synthetic compound 1.

Interestingly, while compound 1 did not affect insulin signaling in murine myoblast C2C12 cells, compound PE/3 showed an evident insulin mimetic activity (Casertano et al., 2022). Based on these results, we recently investigated the influence of two semisynthetic fragments, PE/2 and PE/3, on the insulin sensitivity of hepatocellular carcinoma cell lines (HepG2). Interestingly, remarkable differences in the effects of the two PE fragments were observed. Indeed, only compound PE/2 acted as an insulin-sensitizing agent, increasing insulin-induced INSR/Akt phosphorylation, similar to phosphoeleganin. However, it was not able to inhibit PTP1B. On the contrary, the phosphorylated fragment PE/3, previously shown to be an insulin mimetic agent in muscle cells by significantly inhibiting PTP1B (Casertano et al., 2022), did not improve insulin signaling in HepG2 cells (Agognon et al., 2024). In addition, as PE/2, but not PE/3, decreased IL-6 secretion and expression, we hypothesized that the improvement of hepatic insulin signaling induced by this compound could be mediated by inhibition of this cytokine (Agognon et al., 2024).

In this study, we have investigated the role of the semisynthetic compound PE/2 in crosstalk between hepatocytes and adipocytes by using the conditioned media (CM) approach. Our results suggest that PE/2 affects the liver–adipose axis, in turn improving inflammatory cytokine balance, lipid metabolism, insulin signaling, and glucose utilization in differentiated 3T3-L1 cells.

## 2 Materials and methods

### 2.1 Materials

Cell culture media, sera, and antibiotics were purchased from Invitrogen (Grand Island, NY, USA). Protein electrophoresis, ECL reagents, and real-time PCR reagents were purchased from Bio-Rad (Richmond, VA, USA). Sigma-Aldrich (Saint Louis, MO, USA) provided solvents, deuterated solvents, Oil Red O stain, and all other chemicals. Recombinant IL-6 and LY294002 were purchased from Cell Guidance Systems LLC (St. Louis, MA, USA) and Sigma-Aldrich (St. Louis, MO, USA), respectively. All 1H NMR (700 MHz) analyses were carried out on a Bruker Avance Neo spectrometer (Bruker BioSpin Corporation, Billerica, MA, USA); chemical shifts were referenced to the residual solvent signal (CH_3_OH: δ_H_ = 3.31, δ_C_ = 49.0; CH_3_Cl_3_: δ_H_ = 7.26, δ_C_ = 77.0).

The high-resolution MS spectra (both positive and negative mode) were acquired on a Thermo LTQ Orbitrap XL mass spectrometer (Thermo-Fisher, San Josè, CA, USA). MeOH was used as a solvent for the infusion. High-performance liquid chromatography (HPLC) analyses were performed on a Knauer Azura Pump 4.1 S instrument equipped with a Knauer K-2301 RI detector (LabService Analytica s.r.l., Anzola dell’Emilia, Italy).

### 2.2 Extraction and isolation of PE and semisynthesis of PE/2 by oxidative cleavage of PE

Small specimens of the solitary ascidian *A. elegans* were collected in April 2023 along the coast of Pozzuoli, Italy (40°46′40.0″ N, 14°05′19.0″ E). Following collection, specimens of *A. elegans* were immediately frozen and maintained at low temperature until use. After thawing, the material (28.3 g dry weight after extraction) was extracted following the procedure previously described by our group (Luciano et al., 2017). The butanol-soluble fraction was separated by MPLC on a C18 column with gradient elution using H_2_O, MeOH, and CHCl_3_. Subsequent purification of the MeOH/H_2_O 7:3 (*v/v*) fraction by reversed-phase HPLC (Synergi RP-MAX, 4 μm, 250 × 4.6 mm; MeOH/H_2_O 8:2, *v/v*, supplemented with 0.1% TFA) afforded pure PE (20.2 mg, RT = 20.4 min). PE was isolated as a colorless oil, and all its analytical data were consistent with those previously reported (Luciano et al., 2017). PE/2 was obtained by semisynthetic modification of the marine natural product PE through oxidative cleavage of its 1,2-diol functionality (Imperatore et al., 2016; Luciano et al., 2017). For this purpose, a portion of PE (12.4 mg) was treated with 2.0 mL of 0.01 M NaIO_4_ in methanol under magnetic stirring at room temperature for 3 h. The reaction mixture was subsequently cooled to 0 °C, and a large excess of NaBH_4_ (8.5 mg) was added. After 1 h of additional stirring, the mixture was brought back to room temperature and partitioned twice between water and butanol (2 × 20 mL). The combined organic extracts were dried over Na_2_SO_4_, filtered, and evaporated under reduced pressure. Final purification of the crude residue by RP-HPLC (Luna C18, 3 μm; H_2_O/MeOH 2:8, *v/v*, with 0.1% TFA; flow rate 0.5 mL/min) afforded PE/2 (4.1 mg, RT = 2.3 min) and PE/3 (5.5 mg, RT = 20.3 min) as pure compounds. Their structures were confirmed by spectroscopic analysis and comparison with literature data (Imperatore et al., 2016; Luciano et al., 2017).

### 2.3 Cell culture procedures and differentiation

3T3-L1 mouse embryonic fibroblasts (ATCC-CL-173, American Type Culture Collection, Manassas, VA) were cultivated at 37 °C in a humidified 95% air and 5% CO_2_ atmosphere (all *v/v*) and grown in Dulbecco’s modified Eagle’s medium (DMEM) supplemented with 10% calf serum (CS), 10,000 units/mL penicillin, and 10,000 µg/mL streptomycin. To differentiate 3T3-L1 preadipocytes into adipocytes, cells were seeded at 2.0 × 10^5^/well in a 12-well plate and incubated with the first differentiation mix (DMEM 10% fetal bovine serum (FBS)), containing insulin 870 nM, dexamethasone 10 μM, rosiglitazone 1 μM, and 3-isobutyl-1-methylxanthine 0,5 mM to initiate adipogenesis (day 0). The media were changed every 48 h with the second differentiation mix (DMEM 10% FBS with insulin 870 nM) until mature adipocytes were obtained (day 8). Lipid accumulation and vacuole formation were monitored and imaged using an Olympus IX51 light microscope at ×10 magnification equipped with an Olympus DP23 digital camera and DP2-AOU 1.2 software. To evaluate insulin signaling and cytokine expression, differentiated 3T3-L1 cells were incubated with conditioned media derived from HEPG2 cells for 18 h, while to measure Oil Red O staining and adipogenic gene expression, 3T3-L1 preadipocytes were incubated with conditioned media for 8 days.

### 2.4 Western blot analysis

3T3-L1 adipocytes were lysed with lysis buffer containing 50 mM HEPES, 150 mM MgCl2, 150 mM NaCl, 5 mM EGTA, 1% (*v/v*) Triton X-100, 1% (*v/v*) glycerol, and phenylmethylsulphonyl fluoride (PMSF) (10 µL/mL) and trypsin inhibitor (10 µL/mL). Lysates were centrifuged at 14,000 rpm for 20 min at 4 °C. The protein concentrations in the cell lysates were measured using a Beckman Coulter spectrophotometer (DU530, Life Science UV/VIS) and the Bradford assay by Bio-Rad (Catalog No.5000006, Bio-Rad, Hercules, CA, USA). Proteins (50 µg) were separated by dodecyl sulfate-polyacrylamide gel electrophoresis (SDS-PAGE), transferred to a nitrocellulose membrane, and blocked with 5% BSA in Tris-buffered saline containing 1% Tween-20. Next, the membranes were incubated with specific primary antibodies: pJNK (1:500, Catalog no.: sc-6254, Santa Cruz Biotechnology, Dallas, TX, United States); JNK (1:500, Catalog no.: sc-571, Santa Cruz Biotechnology, Dallas, TX, United States); INSR (1:1,000, catalog no.: sc-713, Santa Cruz Biotechnology, Dallas, TX, United States); pINSR (1:1,000, catalog no.: 3021, Cell Signaling Technology, Beverly, MA, USA); Akt (1:500, catalog no.: 9272, Cell Signaling Technology, Beverly, MA, USA); pAkt (1:500, catalog no.: 9271, Cell Signaling Technology, Beverly, MA, USA); Vinculin (1:1,000, Catalog No: sc-73614, Santa Cruz Biotechnology, Dallas, TX, United States). Secondary antibodies used included anti-rabbit (1:1,000) (catalog code 170-6515, Bio-Rad, Hercules, CA) and anti-mouse antibodies (1:1,000) (catalog code 170-6516, Bio-Rad, Hercules, CA, USA). Where necessary, membranes were subjected to stripping using a Blot stripping buffer (Thermo Scientific, USA) for 15 min, thoroughly washed, and re-blocked before being reprobed with a different primary antibody. Signal detection was performed by chemiluminescence (ECL), and images were acquired using the ChemiDoc system (Bio-Rad Hercules, CA, USA).

### 2.5 Real-time RT-PCR analysis

Total cellular RNA was isolated from 3T3-L1 cells using Qiazol reagent (QIAGEN Sciences, Hilden, Germany). Subsequently, 1 µg of cellular RNA was reverse transcribed using Superscript III reverse transcriptase (Life Technologies, Carlsbad, CA, USA). PCR reactions were analyzed using IQTM SYBR Green Supermix (Bio-Rad, Hercules, CA, USA). Analysis was performed using Platinum SYBR Green qPCR SuperMix-UDG on an iCycler iQ Multicolor real-time PCR detection system (Bio-Rad, Hercules, CA, USA), for a total of 40 cycles. Reactions were then analyzed by normalizing for the arithmetic mean of the Cyclophilin A housekeeping gene and using methods 2^−ΔΔCT^ and 2^−ΔΔCT^. The primer sequences used in this study are reported in the Supplementary Materials section.

### 2.6 Oil Red O staining (ORO)

To evaluate lipid accumulation, 3T3-L1 preadipocytes were incubated with conditioned media. To evaluate differentiation efficiency, Oil Red O (ORO) staining was performed on both undifferentiated control cells and differentiated adipocytes after 8 days of adipogenic induction. Undifferentiated cells were stained using the same protocol to establish a baseline control, allowing us to accurately quantify the increase in the percentage of ORO-positive stained area (%Area) resulting from intracellular lipid droplet accumulation upon differentiation. Microscopic images were captured from at least 3 randomly selected fields per condition and processed using ImageJ software, where binary masks were created by thresholding to isolate ORO-positive lipid droplets relative to the total image field. On day 8 post-differentiation, adipocytes were washed with a phosphate-buffered saline (PBS) solution and fixed with 4% paraformaldehyde for 10 min. Next, cells were washed twice with PBS for 5 min, followed by a brief wash with 60% isopropanol, and then stained with Oil Red O (ORO) solution (0.5 g working solution of ORO powder dissolved in 60% ethanol) for 30 min at room temperature. After washing with PBS, the cells were analyzed by light microscopy and subsequently photographed with one microscopic field (10X). The percentage of lipid deposition was quantified using color histogram analysis in ImageJ software (v 1.54p NIH, USA) (Deutsch M.J). Once the image scale was set (µm), analysis was performed by applying color deconvolution and converting the RGB images to 8-bit grayscale, followed by thresholding to generate a binary mask representing the lipid droplets to calculate the percentage of stained area (%Area).

### 2.7 Analysis of glucose consumption

Extracellular glucose concentrations were determined in adipocytes using the Piccolo Xpress® automated clinical chemistry analyzer (Abaxis, Union City, CA, USA) with the Amlyte 13 reagent panel and Lipid Panel Plus. The assay is based on a coupled enzymatic reaction involving hexokinase and glucose-6-phosphate dehydrogenase (HK/G6PDH), with photometric detection of NADH production at 340 nm. Given that the system was employed on a non-standard matrix (culture medium), accuracy was previously validated by testing an aliquot of unconditioned DMEM, natively supplemented with 4,5 g/L D-glucose. The measured value of 454 mg/dL confirmed the high precision of the system and the absence of significant matrix effects or interference from medium components. A critical aspect of our validation concerned the potential interference of phenol red, a pH indicator ubiquitous in cell culture media. Phenol red exhibits significant light absorption at wavelengths (typically 500–550 nm) that overlap with the spectrophotometric range used in many enzymatic glucose assays (such as those based on glucose oxidase or hexokinase reactions).

For the metabolic assays, conditioned media were prepared using a medium consisting of high-glucose DMEM supplemented with 0.25% bovine serum albumin, followed by the addition of specific experimental stimuli. To ensure data comparability, all experiments were performed in multi-well plates (MW) with a standardized initial seeding density, ensuring an identical number of cells in each well. 3T3-L1 adipocytes were then incubated with these conditioned media for 18 h. Adipocyte glucose consumption was assessed as a functional metabolic readout of insulin signaling. Since the baseline glucose concentration depends on the first incubation with hepatocytes, we measured the glucose level of each CM (CM, CM_Ins_, and CM_Ins+PE/2_) immediately before transferring it to the 3T3-L1 cultures (*G*_*initial*_). Following 18-h incubation, the remaining glucose in the supernatant (*G*_*final*_) was determined. Net adipocyte glucose consumption (Δ*Glucose*) was calculated as *G*_*initial*_ *- G*_*final*_. In parallel, background glucose stability and evaporation were monitored using cell-free wells incubated under identical experimental conditions. Final values were normalized for protein content by the Bio-Rad DC assay.

### 2.8 Statistical analysis

Data were analyzed with GraphPad Prism 10.4.0 (GraphPad Inc., San Diego, CA, USA). Multiple comparisons among more than two groups were made using the ANOVA test with Tukey correction (for normally distributed data). P values equal to or less than 0.05 were considered statistically significant.

## 3 Results

### 3.1 Re-isolation of PE and oxidative cleavage to PE/2

PE was re-isolated from specimens of the tunicate *A. elegans* collected in the Bay of Pozzuoli by applying the isolation method previously developed in our laboratory, based on exhaustive extraction followed by consecutive chromatographic fractionation and purification steps. A portion of the purified polyketide PE was subsequently submitted to oxidative cleavage, yielding compounds PE/2 and PE/3 (Figure 1). The purity of all compounds was verified by NMR analysis, and their identity was unequivocally confirmed by matching their spectroscopic data with those previously reported in the literature (Imperatore et al., 2016; Luciano et al., 2017).

### 3.2 Effect of CM from PE/2-treated HepG2 cells on cytokine balance in 3T3-L1 adipocytes

The impact of CM derived from PE/2-treated HepG2 cells (CM_PE/2_) on the inflammatory cytokine profile of differentiated 3T3-L1 adipocytes was evaluated by measuring the expression levels of *adiponectin*, having anti-inflammatory properties, and of pro-inflammatory cytokines, such as *IL-8*, *IL-1β*, *TNFα*, and *IL-6*. As shown in Figure 2A, CM_PE/2_ increased *adiponectin* mRNA by 1.85-fold compared to HepG2-derived CM alone. In parallel, CM_PE/2_ decreased the mRNA levels of *IL-8* and *IL-1β* by 43% and 52%, respectively (Figures 2B,C), without affecting *TNFα* and *IL-6* expression (Figures 2D,E). In parallel, the phosphorylation of JNK, one of the main mediators of cytokine-induced inflammation, decreased by 36% in PE/2-treated HepG2 cells compared to cells incubated with CM (Figure 2F).

**FIGURE 2:**
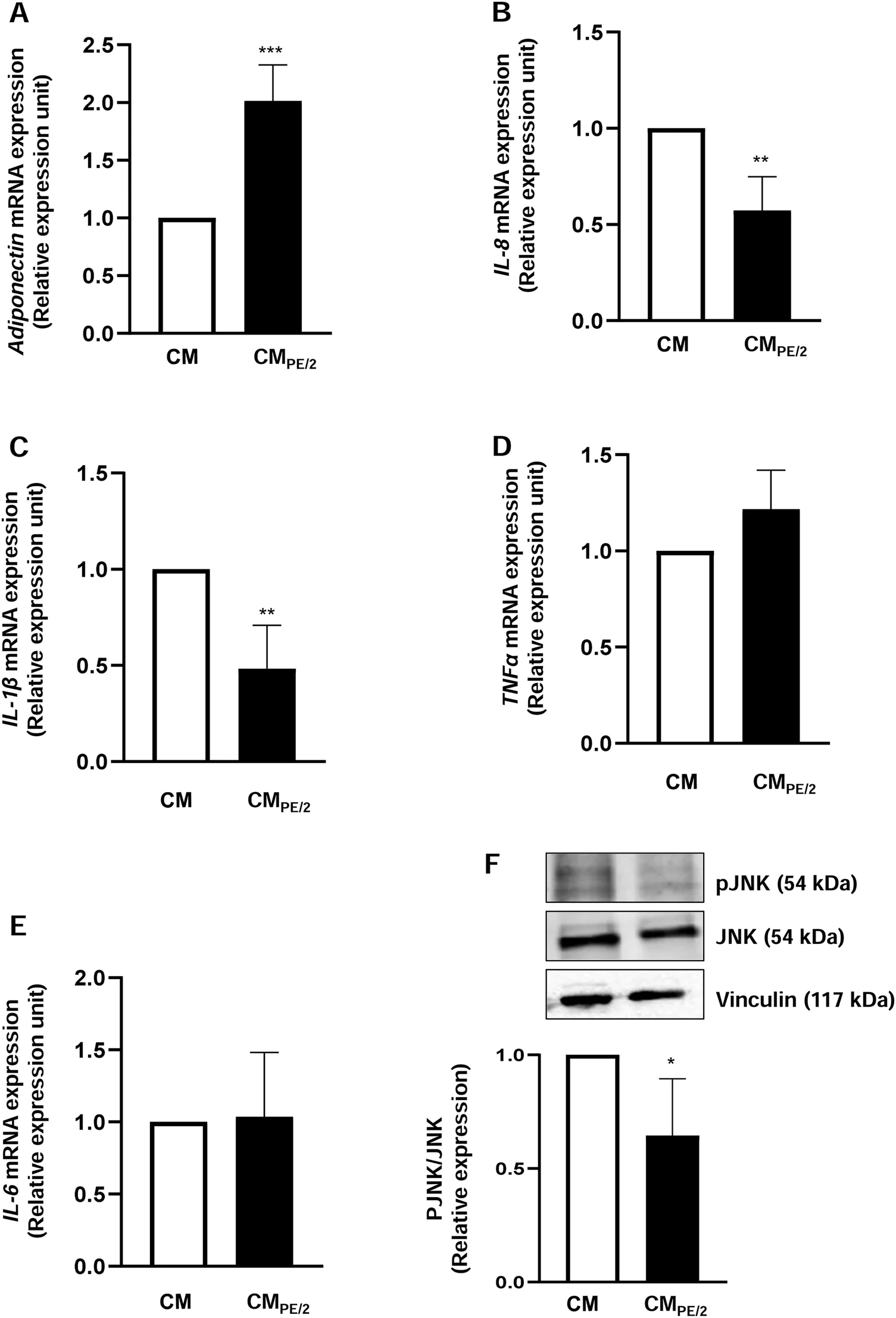
Effect of conditioned media from HepG2 cells on the balance of inflammatory cytokines in 3T3-L1 cells. Differentiated 3T3L1 cells were incubated for 18 h with HepG2-derived conditioned medium (CM) alone or CM from HepG2 cells incubated with PE/2 (25 μM, 4 h) (CM_PE/2_). *Adiponectin* **(A)**, *IL-8* **(B)**, *IL1β* **(C)**, *TNFα* **(D)**, and *IL-6* **(E)** mRNA levels were evaluated by real-time RT-PCR. Data were normalized to the amount of *Cyclophilin A*, used as an internal control. Bars represent the mean ± SD of n = 3 independent biological replicates, each performed in technical triplicate. **(F)** The protein expression of pJNK and JNK was evaluated by Western blotting. Vinculin bands were taken from a parallel gel loaded with the same lysates. The autoradiographs shown are representative of three different experiments and were subjected to densitometric analysis. Bars represent the mean ± SD of n = 3 independent experiments. Normality of data distribution and homogeneity of variances were verified using the Shapiro-Wilk test. Statistical significance between groups was analyzed using a two-tailed paired t-test (*p < 0.05; **p < 0.01; ***p < 0.001).

### 3.3 Effect of CM from PE/2-treated HepG2 cells on lipid metabolism and adipogenesis

To elucidate the effect of CM_PE/2_ on lipid accumulation, adipocytes were stained with Oil Red O and visualized under a microscope. The amount of lipid droplets was 1.75-fold higher in adipocytes incubated with CM_PE/2_ than in those incubated with CM (Figure 3A). Surprisingly, mRNA levels of *Sterol-Regulatory Element-Binding Protein 1c* (*SREBP1c*) and *Carbohydrate Response Element-Binding Protein* (*ChREBP*), which play a crucial role in regulating lipid synthesis, and their downstream effector *Fatty Acid Synthase* (*FAS*) did not change in the presence of CM_PE/2_ (Figures 3B–D). However, when we evaluated adipocyte differentiation by measuring the expression of the main adipogenic transcription factors, we observed that CM_PE/2_ increased the expression of *Peroxisome Proliferator-Activated Receptor gamma* (*PPARγ*), *CCAAT enhancer binding proteins α* (*C/EBPα*), *fatty acid binding protein 4* (*FABP4*), and *glucose transporter type 4* (*GLUT4*) by 1.59-, 2.78-, 3.90-, and 2.63-fold, respectively (Figures 4 A–D).

**FIGURE 3:**
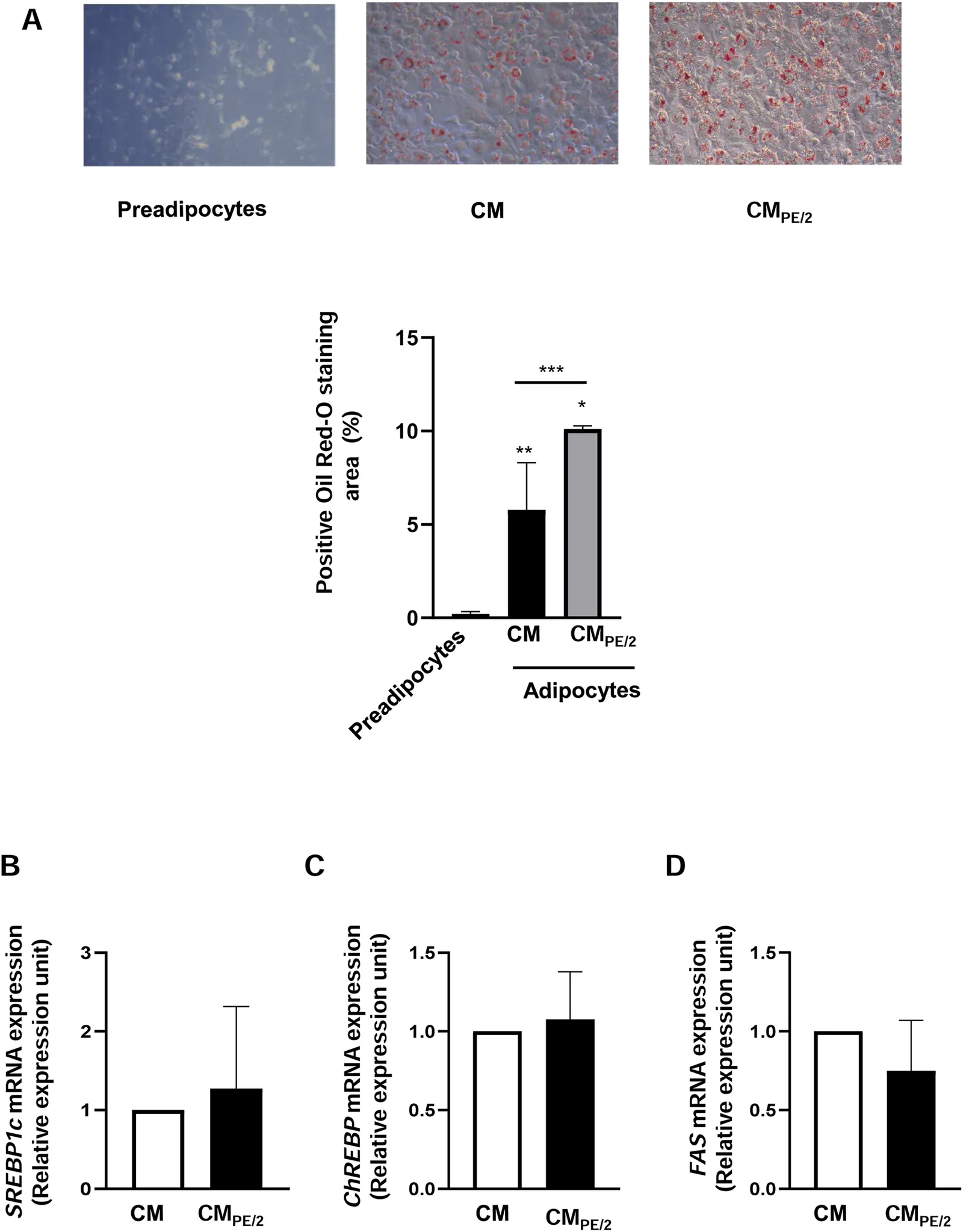
Effect of conditioned media from HepG2 cells on lipid accumulation and lipogenesis in 3T3-L1 cells. **(A)** 3T3-L1 preadipocytes were incubated with HepG2-derived conditioned medium (CM) alone or CM from HepG2 cells incubated with PE/2 (25 μM, 4 h) (CM_PE/2_), and stained with Oil Red O on day 8 post-differentiation. The photographs shown are representative of three different experiments. Normality of data distribution and homogeneity of variances were verified using the Shapiro-Wilk test. Statistical significance between groups was analyzed using ordinary one-way ANOVA followed by Tukey’s multiple comparisons test (*p < 0.05; **p < 0.01; ***p < 0.001). *SREBP1c* **(B)**, *ChREBP* **(C)**, and *FAS* **(D)** mRNA levels were evaluated by real-time RT-PCR. Data were normalized to the amount of *Cyclophilin A*, used as an internal control. Bars represent the mean ± SD of n = 3 independent biological replicates, each performed in technical triplicate. Normality of data distribution and homogeneity of variances were verified using the Shapiro-Wilk test.

**FIGURE 4:**
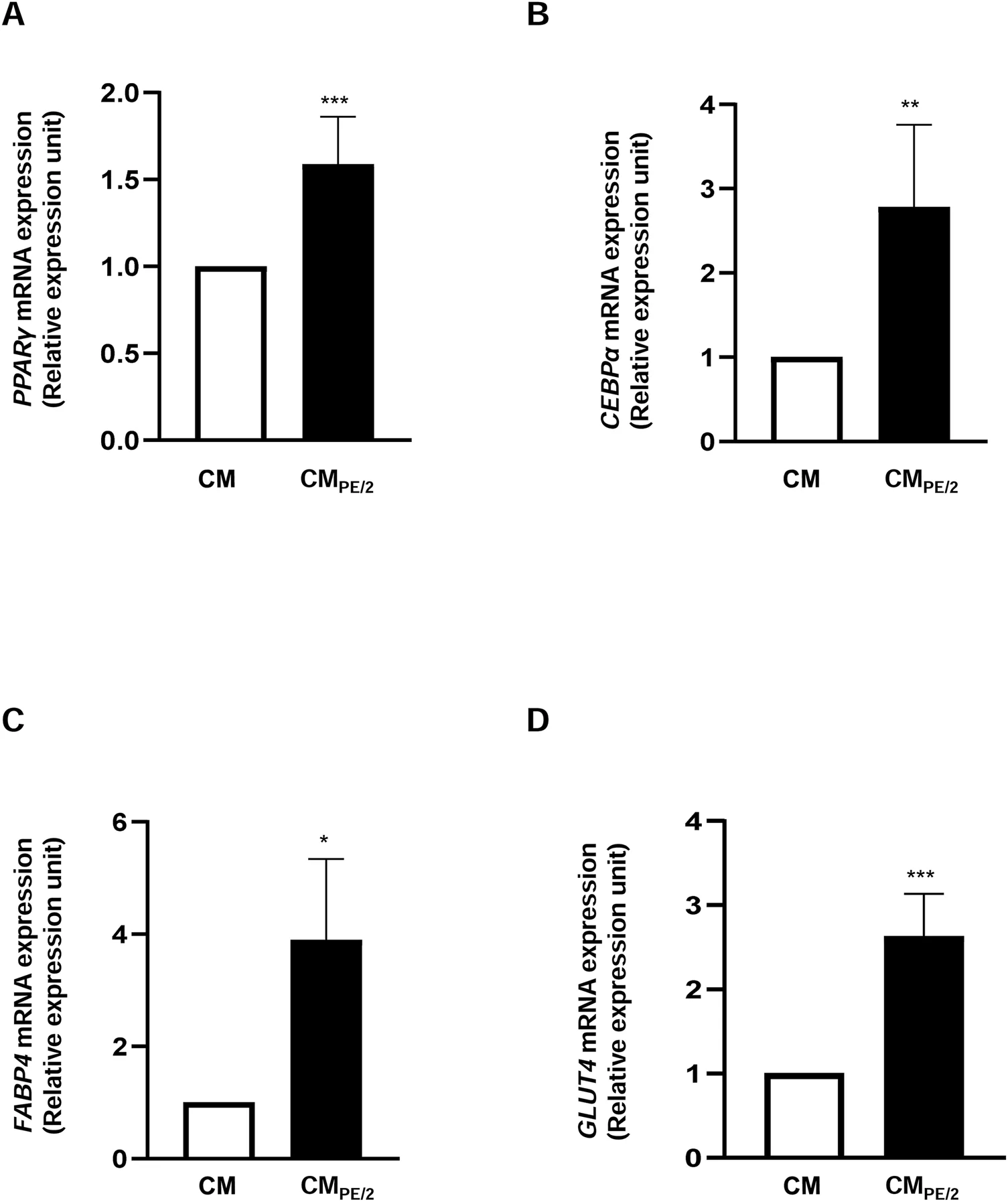
Effect of conditioned media from HepG2 cells on adipogenesis in 3T3-L1 cells. 3T3-L1 preadipocytes were incubated with HepG2-derived conditioned medium (CM) alone or CM from HepG2 cells incubated with PE/2 (25 μM, 4 h) (CM_PE/2_), and *PPARγ* **(A)**, *C/EBPα* **(B)**, *FABP4* **(C)**, and *GLUT4* **(D)** mRNA levels were evaluated by real-time RT-PCR on day 8 post-differentiation. Data were normalized to the amount of *Cyclophilin A*, used as an internal control. Bars represent the mean ± SD of n = 3 independent biological replicates, each performed in technical triplicate. Normality of data distribution and homogeneity of variances were verified using the Shapiro-Wilk test. Statistical significance between groups was analyzed using a two-tailed paired t-test (*p < 0.05; **p < 0.01; ***p < 0.001).

### 3.4 Effect of CM from PE/2-treated HepG2 cells on the insulin signaling in 3T3-L1 adipocytes

To investigate the effect of PE/2 on the insulin pathway in adipocytes, 3T3L1 cells were differentiated and incubated with CM from HepG2 cells treated with insulin alone (CM_Ins_) or with insulin and PE/2 (CM_Ins+PE/2_). As shown in Figure 5A, insulin receptor tyrosine phosphorylation (pINSR) and Akt phosphorylation did not significantly increase in the presence of CM_Ins_. In contrast, CM_Ins+PE/2_ stimulated INSR and Akt phosphorylation by 2.13- and 1.63-fold, respectively, compared to CM, and 1.83- and 1.42-fold compared to CM_Ins_. Total levels of INSR and Akt did not change in the presence of CM_Ins_ and CM_Ins+PE/2_.

**FIGURE 5:**
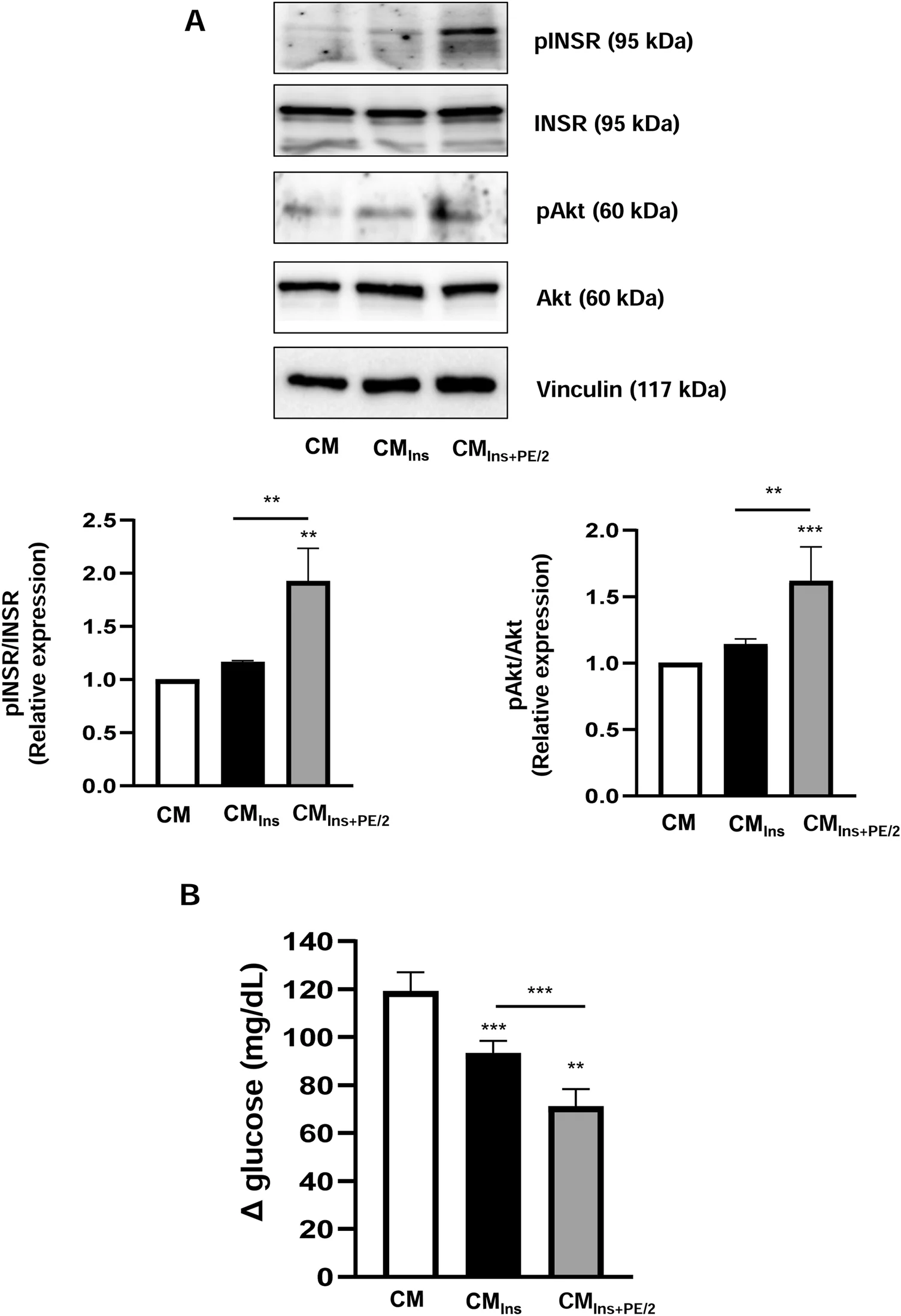
Effect of conditioned media from HepG2 cells on insulin receptor (INSR) and Akt phosphorylation and glucose consumption in 3T3-L1 cells. Differentiated 3T3-L1 cells were incubated for 18 h with HepG2-derived conditioned medium (CM) alone, CM from HepG2 cells stimulated with insulin (100 nM, 10 min) (CM_Ins_), and CM from HepG2 cells incubated with PE/2 (25 μM, 4 h) and stimulated with insulin (100 nM, 10 min) (CM_Ins+PE/2_). **(A)** The protein expression of pINSR, INSR, pAkt, and Akt was evaluated by Western blotting. Vinculin bands were taken from a parallel gel loaded with the same lysates. The autoradiographs shown are representative of three different experiments and were subjected to densitometric analysis. Bars represent the mean ± SD of n = 3 independent experiments. Normality of data distribution and homogeneity of variances were verified using the Shapiro-Wilk test. Statistical significance between groups was analyzed using ordinary one-way ANOVA followed by Tukey’s multiple comparisons test (**p < 0.01; ***p < 0.001). **(B)** Glucose consumption was evaluated by measuring medium glucose concentrations in 3T3-L1 adipocytes, and net glucose uptake (Δ Glucose) was determined by subtracting the remaining glucose concentration in the supernatant (G_final_) from the pre-transfer baseline concentration (G_initial_). Bars represent the mean ± SD of three independent experiments. Normality of data distribution and homogeneity of variances were verified using the Shapiro-Wilk test. Statistical significance between groups was analyzed using ordinary one-way ANOVA followed by Tukey’s multiple comparisons test (**p < 0.01; ***p < 0.001).

As a functional outcome of insulin pathway activation, we evaluated the CM effect on glucose consumption. Medium glucose levels were 22% and 40% lower in the presence of CM_Ins_ and CM_Ins+PE/2_ than in CM, respectively. Interestingly, CM_Ins+PE/2_ decreased the medium glucose concentration by 24% compared with CM_Ins_, reflecting an increased consumption rate (Figure 5B). We did not consider the effect of CM from HepG2 cells stimulated with PE/2 alone, as we had previously shown that PE/2 did not affect insulin signaling in the absence of insulin (Agognon et al., 2024).

To evaluate the importance of Akt signaling in our observed phenotype, 3T3L1 cells were incubated with CM from HepG2 cells treated with insulin, PE/2 plus LY294002 (CM_Ins+PE/2+LY294002_), a widely used inhibitor for blocking Akt (Miele et al., 2007; Yang et al., 2013; de Oliveira et al., 2019). As shown in Figure 6A, the CM_Ins+PE/2_-mediated increase in pAkt levels was completely blocked by LY294002 co-treatment. In parallel, CM_Ins+PE/2+LY294002_ increased glucose levels by 1.55-fold (Figure 6B) and reduced lipid accumulation by 51% (Figure 6C) compared to CM_Ins+PE/2_, restoring both parameters to levels comparable to the CM control.

**FIGURE 6:**
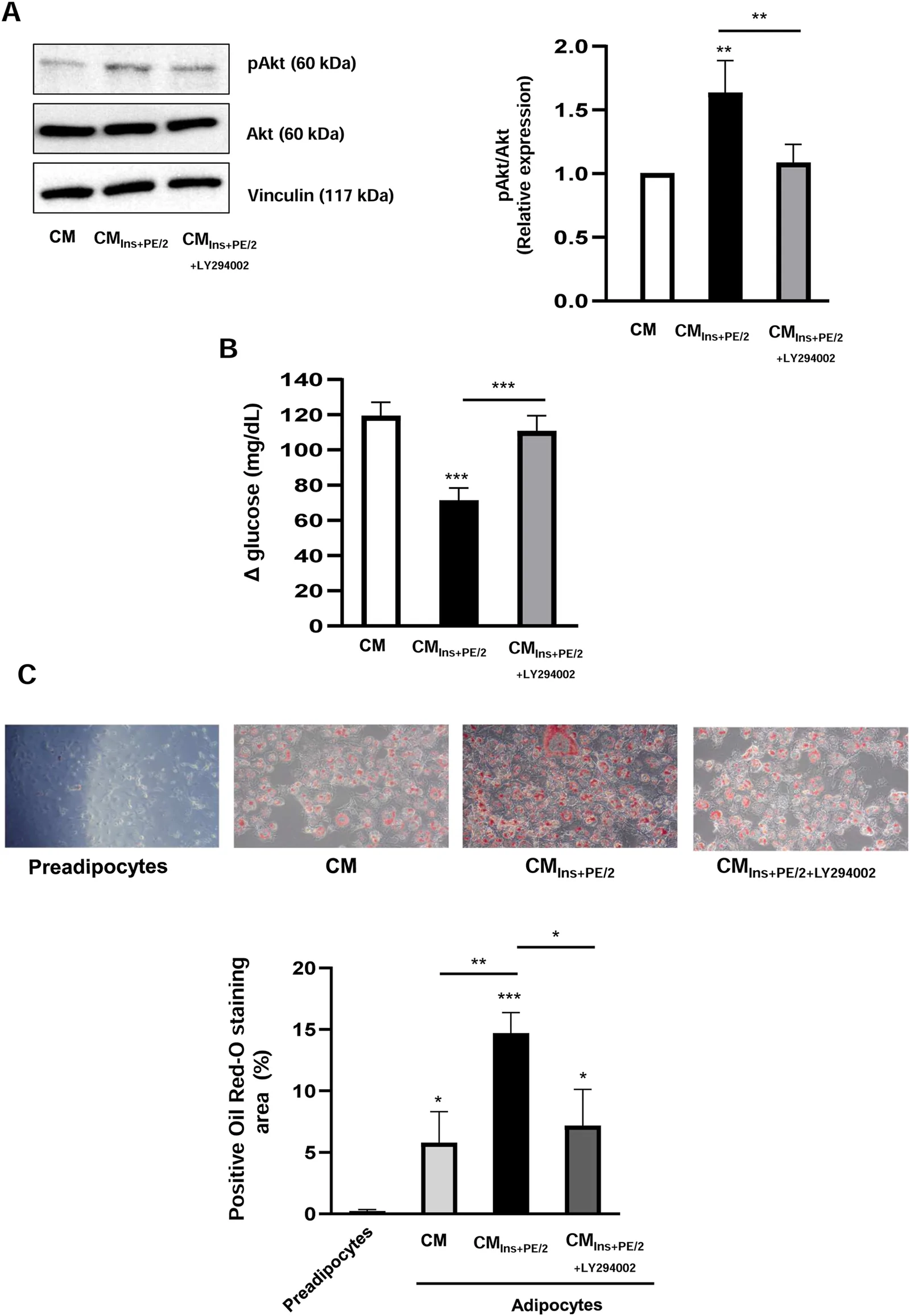
Effect of LY294020 on CM_Ins+PE/2_-mediated signaling. **(A)** Differentiated 3T3-L1 cells were incubated for 18 h with HepG2-derived conditioned medium (CM) alone, CM from HepG2 cells incubated with PE/2 (25 μM, 4 h), stimulated with insulin (100 nM, 10 min) (CM_Ins+PE/2_), and CM from HepG2 cells incubated with PE/2 (25 μM, 4 h), LY294002 (15 μM, 30 min), and stimulated with insulin (100 nM, 10 min) (CM_Ins+PE/2+LY294002_). The protein expression of pAkt and Akt was evaluated by Western blotting. Vinculin bands were taken from a parallel gel loaded with the same lysates. The autoradiographs shown are representative of three different experiments and were subjected to densitometric analysis. Bars represent the mean ± SD of n = 3 independent experiments. **(B)** Glucose consumption was evaluated by measuring medium glucose concentrations in 3T3-L1 adipocytes, and net glucose uptake (Δ Glucose) was determined by subtracting the remaining glucose concentration in the supernatant (G_final_) from the pre-transfer baseline concentration (G_initial_). Bars represent the mean ± SD of three independent experiments. **(C)** 3T3-L1 preadipocytes were incubated for 18 h with HepG2-derived conditioned medium (CM) alone, CM from HepG2 cells incubated with PE/2 (25 μM, 4 h), stimulated with insulin (100 nM, 10 min) (CM_Ins+PE/2_), and CM from HepG2 cells incubated with PE/2 (25 μM, 4 h), LY294002 (15 μM, 30 min), and stimulated with insulin (100 nM, 10 min) (CM_Ins+PE/2+LY294002_), and stained with Oil Red-O on day 8 post-differentiation. The photographs shown are representative of three different experiments. Normality of data distribution and homogeneity of variances were verified using the Shapiro-Wilk test. Statistical significance between groups was analyzed using ordinary one-way ANOVA followed by Tukey’s multiple comparisons test (*p < 0.05; **p < 0.01; ***p < 0.001).

### 3.5 Effect of recombinant IL-6 on CM_PE/2_-induced 3T3-L1 adipocytes improvement

We have previously hypothesized that the improvement of cytokine balance and metabolism in 3T3-L1 adipocytes could be due to the low levels of IL-6 in the CM_PE/2_ medium. To further investigate this issue, 3T3-L1 adipocytes were incubated with CM from HepG2 cells treated with CM alone (CM), PE/2 (CM_PE/2_), or PE/2 plus IL-6 (CM_PE/2+IL6_), bringing the IL-6 levels back to those measured in the CM. Interestingly, CM_PE/2+IL6_ reduced the expression of *adiponectin* by 40% and increased *IL-8* and *IL-1β* by 1.77-and 2.60-fold, respectively, compared to CM_PE/2_ (Figures 7A–C). Consistent with these data, mRNA levels of *PPARγ*, C*/EBPα*, and *GLUT4* were 47, 53, and 57% lower in 3T3-L1 adipocytes incubated with CM_PE/2+IL6_ than in those treated with CM_PE/2_ (Figures 7D–F). Finally, glucose consumption also returned to control levels in the presence of CM_PE/2+IL6_ (Figure 7G).

**FIGURE 7:**
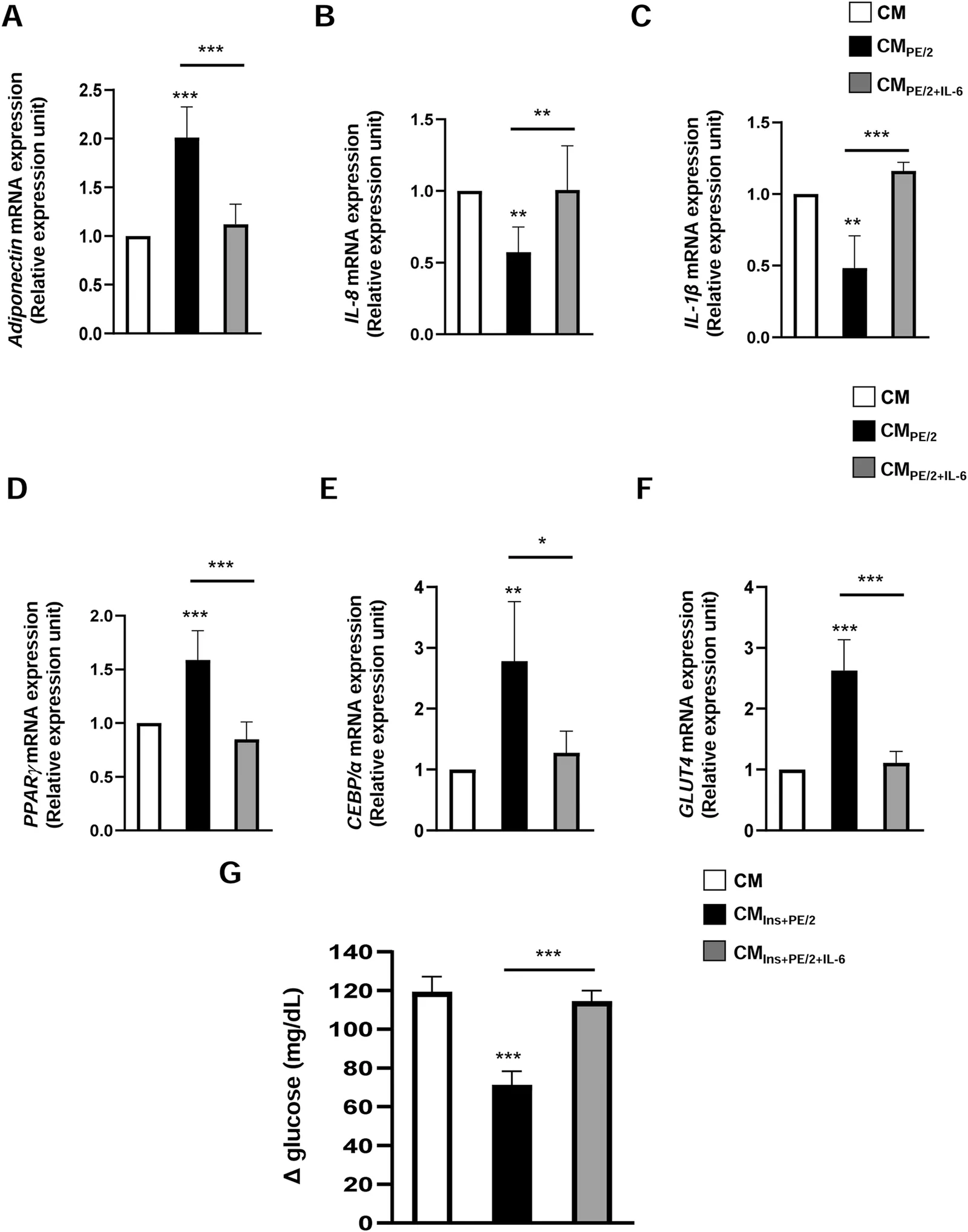
Effect of recombinant IL-6 on CM_Ins+PE/2_-mediated signaling. **(A)** Differentiated 3T3-L1 cells were incubated for 18 h with HepG2-derived conditioned medium (CM) alone, CM from HepG2 cells incubated with PE/2 (25 μM, 4 h) (CM_PE/2_), and CM from HepG2 cells incubated with PE/2 (25 μM, 4 h) and IL-6 (1.65 pg/mL, 4 h) (CM_PE/2+IL-6_). *Adiponectin* **(A)**, *IL-8* **(B)**, and *IL-1β* **(C)** mRNA levels were evaluated by real-time RT-PCR. Data were normalized to the amount of *Cyclophilin A*, used as an internal control. Bars represent the mean ± SD of n = 3 independent biological replicates, each performed in technical triplicate. 3T3-L1 preadipocytes were incubated for 18 h with HepG2-derived conditioned medium (CM) alone, CM from HepG2 cells incubated with PE/2 (25 μM, 4 h) (CM_PE/2_), and CM from HepG2 cells incubated with PE/2 (25 μM, 4 h) and IL-6 (1.65 pg/mL, 4 h) (CM_PE/2+IL-6_), and *PPARγ* **(D)**, *C/EBPα* **(E)**, and *GLUT4* **(F)** mRNA levels were evaluated by real-time RT-PCR on day 8 post-differentiation. Data were normalized to the amount of *Cyclophilin A*, used as an internal control. Bars represent the mean ± SD of n = 3 independent biological replicates, each performed in technical triplicate. **(G)** Differentiated 3T3-L1 cells were incubated for 18 h with HepG2-derived conditioned medium (CM) alone, CM from HepG2 cells incubated with PE/2 (25 μM, 4 h), stimulated with insulin (100 nM, 10 min) (CM_Ins+PE/2_), and CM from HepG2 cells incubated with PE/2 (25 μM, 4 h), IL-6 (1.65 pg/mL, 4 h), and stimulated with insulin (100 nM, 10 min) (CM_Ins+PE/2+IL-6_). Glucose consumption was evaluated by measuring medium glucose concentrations in 3T3-L1 adipocytes, and net glucose uptake (Δ Glucose) was determined by subtracting the remaining glucose concentration in the supernatant (G_final_) from the pre-transfer baseline concentration (G_initial_). Bars represent the mean ± SD of three independent experiments. Normality of data distribution and homogeneity of variances were verified using the Shapiro-Wilk test. Statistical significance between groups was analyzed using ordinary one-way ANOVA followed by Tukey’s multiple comparisons test (*p < 0.05; **p < 0.01; ***p < 0.001).

## 4 Discussion

The liver maintains a dynamic crosstalk with multiple organs, including the adipose tissue, as it secretes several cytokines, hepatokines, and transcription factors that regulate metabolic homeostasis. Proteomic studies have quantified approximately 10.200 proteins synthesized in humans, of which 40% are produced in the liver and secreted (Watt et al., 2019). These molecules exert pleiotropic effects on lipid and glucose metabolism and on insulin action. Importantly, their secretion is altered in pathological conditions such as diabetes, obesity, and MAFLD. For example, the hepatic cytokines TNFα and IL-6 play a pivotal role in the pathogenesis of metabolic diseases in humans (Gonzalez-Amaro et al., 1994; Spencer et al., 2013; Aziel Alvarado-Ojeda et al., 2024; Tshivhase et al., 2024; Zhao and Tian, 2024). On the other hand, adipose tissue also secretes several cytokines, such as adiponectin, that play a crucial role in mitigating inflammation, insulin resistance, and MAFLD progression (Lagathu et al., 2003; Rotter et al., 2003; Fasshauer et al., 2004; Kralisch et al., 2005; Rafaqat et al., 2024).

Currently, in contemporary medicine, the modulation of involved cytokines represents one of the most significant challenges in the treatment of metabolic pathologies, as these conditions not only impair patients’ quality of life but also impose an enormous social and economic burden. In this context, the development of new therapies and the search for new pharmacological molecules play a crucial role.

In the last few years, marine natural products (MNPs) have attracted increasing scientific attention. Marine species are known to produce secondary metabolites with structural features rarely observed in terrestrial organisms. Within this context, the Mediterranean ascidian *A. elegans* has yielded a new phosphorylated polyketide, phosphoeleganin, whose stereochemical properties have been fully characterized (Imperatore et al., 2016; Luciano et al., 2017).

To better investigate the biologically relevant chemical space of PE, the natural metabolite was structurally manipulated to obtain smaller and less complex fragments, designated PE/2 and PE/3, and their effects on insulin responsiveness were assessed in HepG2 cells. The advantage of generating these molecules was that they could be modified and had greater chemical accessibility than PE.

Interestingly, the two fragments exhibited clearly divergent biological behaviors and tissue-specific action. PE/2 was capable of diminishing IL6 expression and secretion and boosting insulin-induced INSR/Akt phosphorylation in hepatocytes. In contrast, PE/3, previously described as an insulin mimetic in muscle cells (Casertano et al., 2022), did not enhance insulin signaling in this cell type (Agognon et al., 2024).

In the current work, we evaluated the potential role of the semisynthetic compound PE/2 in the crosstalk between hepatocytes and adipocytes. To ensure high reproducibility, unlimited availability, and phenotypic stability, HepG2 and 3T3-L1 cells were selected as the donor hepatocyte and recipient adipocyte models. Although they exhibit a metabolic and secretory profile distinct from those of human primary hepatocytes and adipocytes, they successfully overcome the high inter-donor variability and costs associated with primary cells (Poulos et al., 2010; Kotlyarova et al., 2026). The communication between these two cell types was modeled using a conditioned media (CM) approach. This method is highly effective for studying cell-cell interactions because CM contains cytokines, growth factors, extracellular vesicles secreted by the ‘donor’ cells, and mimics paracrine and autocrine communication *in vitro*, allowing us to understand how these soluble factors influence the ‘recipient’ cells.

Our experiments indicate that the expression of *adiponectin*, an adipokine acting as an anti-inflammatory and insulin-sensitizing agent (Hajri et al., 2011), is increased by CM from HepG2 cells incubated with PE/2 (CM_PE/2_) compared to CM alone. On the other hand, *IL-8* and *IL-1β*, known to have pro-inflammatory properties and dysregulatory effects on glucose and lipid metabolism (Lagathu et al., 2006; Kobashi et al., 2009), were significantly decreased by CM_PE/2_. In parallel, the phosphorylation of JNK, a key mediator of inflammation, is reduced in the presence of CM_PE/2_.

A reduced availability of anti-inflammatory adiponectin, combined with elevated pro-inflammatory cytokines, directly impairs adipocyte function and alters intracellular lipid metabolism (Tao et al., 2014; Sukhorukov and Orekhov, 2024). To investigate this effect, we performed ORO staining to visualize intracellular lipids. The findings obtained indicated a moderately greater lipid accumulation in adipocytes incubated with CM_PE/2_ compared to those incubated with CM alone. This finding is interesting since, *in vivo*, a moderate increase (∼2-fold) in adipogenesis does not impair adipose tissue expandability but rather optimizes it, promoting healthy and metabolically protective expansion (Sun et al., 2011).

Since the accumulation of lipid droplets may be due to an increase in lipogenesis and/or adipogenesis, the expression of *SREBP1c* and *ChREBP*, key transcription factors that regulate lipogenesis and its downstream target FAS, was measured.

Interestingly, the mRNA levels of *SREBP1c*, *ChREBP*, and *FAS* did not change following incubation with CM_PE/2_, indicating that lipid synthesis could occur through another pathway that is not directly regulated by these proteins. In addition, Sekiya et al. indicated that, unlike in hepatocytes, SREBP1c is not the master regulator of lipogenesis in adipocytes (Sekiya et al., 2007).

Adipogenesis represents another fundamental process of lipid metabolism and is orchestrated by specific transcription factors, including *PPARγ*, *C/EBPα*, *FABP4*, and *GLUT4*. Notably, all these molecules were upregulated in the presence of CM_PE/2_. The increased levels of *PPARγ* and *C/EBPα* could explain the improved lipid accumulation in 3T3-L1 cells incubated with CM_PE/2_, independently of canonical *de novo* lipogenesis pathways. Indeed, both transcription factors promote triglyceride uptake and storage, as indicated by several authors (Tamori et al., 2002; Matsusue et al., 2004; Zhang et al., 2004; Kang et al., 2015; Satoh et al., 2019). Furthermore, the concomitant increase in FABP4 expression could facilitate intracellular fatty acid transport and trafficking, directly promoting lipid droplet growth and triglyceride storage (Gan et al., 2015).

Compelling evidence links cytokines and chemokines to the pathogenesis of insulin resistance in adipocytes (Hotamisligil et al., 1993; Rotter et al., 2003; Jager et al., 2007). Interestingly, CM from HepG2 cells treated with insulin and PE/2 (CM_Ins+PE/2_) increases INSR and Akt phosphorylation and glucose utilization in differentiated 3T3-L1 cells compared with control (CM) and insulin alone (CM_Ins_). Treatment with CM containing the PI3K/Akt inhibitor LY294002 significantly reversed the cellular improvements induced by CM_Ins+PE/2_, indicating involvement of this signaling pathway.

Since we previously demonstrated a 26% reduction in IL-6 levels in the culture medium of HepG2 cells treated with PE/2 compared to the control (Agognon et al., 2024), we hypothesized that the lower IL-6 concentrations in CM_PE/2_ could improve cytokine balance and metabolism in 3T3-L1 adipocytes. To address this issue, we incubated 3T3-L1 adipocytes with CM_PE/2_ supplemented with recombinant IL-6, restoring its concentration to the levels observed in the CM. Notably, we observed a significant reduction in the protective effect of PE/2, with the levels of adiponectin, proinflammatory cytokines, lipogenesis, and glucose consumption returning to values similar to those of the control medium. This result corroborates the involvement of IL-6 in the mechanism of action of PE/2 and is particularly interesting. Indeed, IL-6 plays a multi-faceted role in adipose tissue pathophysiology. Fasshauer et al. reported that IL-6 suppresses adiponectin expression (Fasshauer et al., 2003), while Okamura et al. and Minogue et al. showed that IL-6 induces an upregulation of IL-8 and IL-1β, respectively (Minogue et al., 2012; Okamura et al., 2013). Furthermore, several authors have shown that IL-6 contributes to the etiology of insulin resistance, at least in 3T3-L1 cells (Lagathu et al., 2003; Rotter et al., 2003; Fasshauer et al., 2004; Kralisch et al., 2005), and inhibits both adipogenesis and lipogenesis (Almuraikhy et al., 2016; Radvanyi and Roszer, 2024).

In summary, our findings suggest that CM_PE/2_, characterized by low levels of IL-6, ameliorates cytokine balance as well as lipid and glucose metabolism in 3T3-L1 adipocytes (Figure 8), thereby providing novel insights into the cellular mechanisms of the polyketide PE/2 and its relevance to metabolic diseases. However, these findings require independent validation in primary cell systems and *in vivo* models. The limited availability of PE/2, a semisynthetic derivative of a marine-derived natural product, currently constrains further experimental validation, while its very low solubility in water and physiological saline necessitates formulation development before *in vivo* evaluation. Furthermore, whether PE/2 modulates other key hepatokines and whether these contribute to the effects of the conditioned medium remain to be investigated.

**FIGURE 8:**
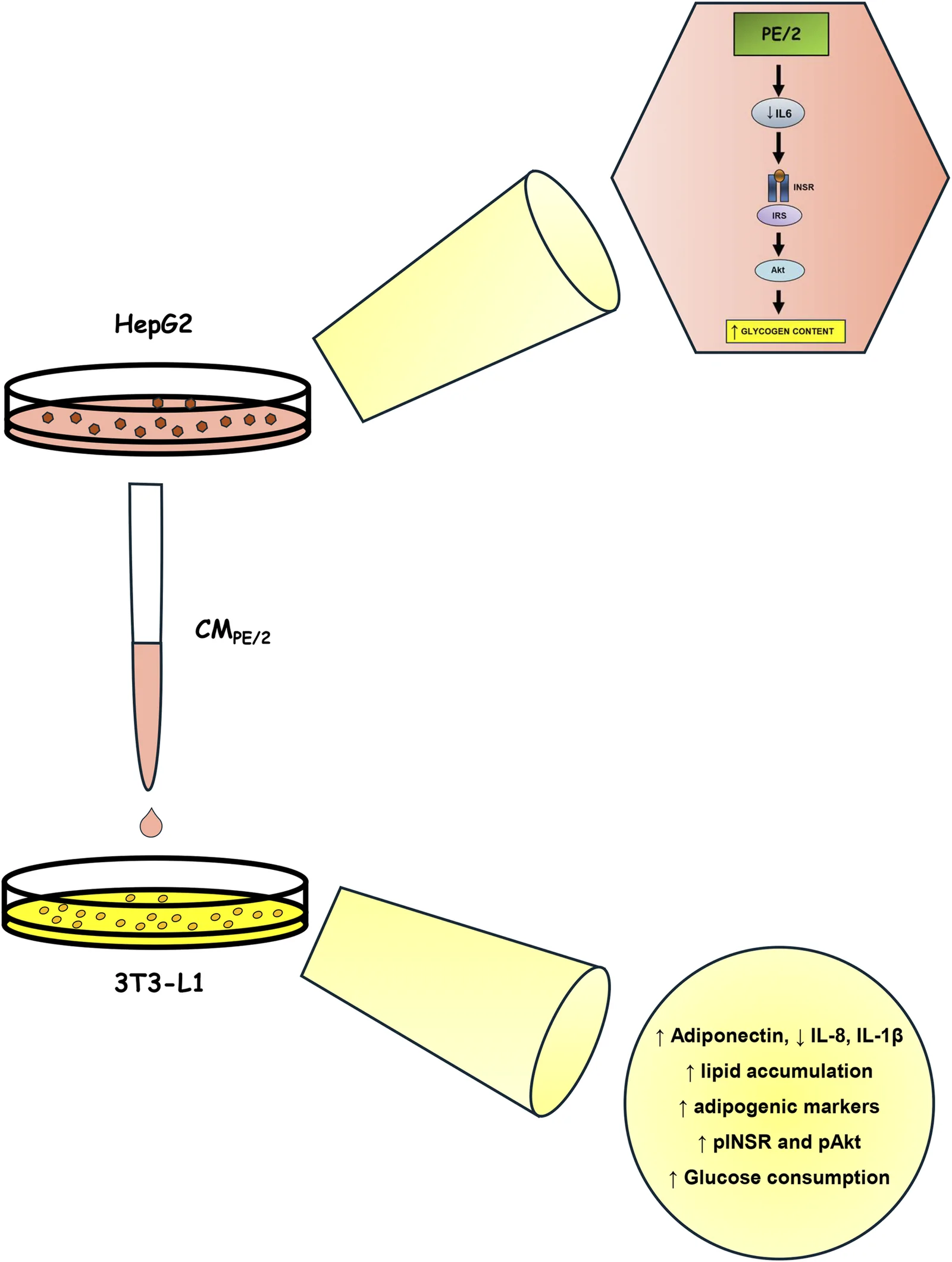
Summary of the effects of CM_PE/2_ on 3T3-L1 cells. PE/2, by reducing IL-6, increases insulin signaling in HepG2 cells (red hexagon). CM from HepG2 cells incubated with PE/2 (CM_PE/2_), characterized by low IL6 levels, improves cytokine balance, promotes lipid accumulation, and increases adipogenic marker expression and insulin signaling in 3T3-L1 adipocytes (yellow circle).

## Data Availability

The raw data supporting the conclusions of this article will be made available by the authors, without undue reservation.
