# Supplementary material for: Conditioned media derived from HepG2 cells treated with phosphoeleganin synthetic-derived compound PE/2 improve cytokine balance and metabolic response in 3T3-L1 adipocytes
Source: Front Pharmacol. 2026 Oct 23;17:1909848. doi: 10.3389/fphar.2026.1909848 (PMC13739515; doi:10.3389/fphar.2026.1909848)
Supplement: Supplementary file 1 [file DataSheet1.pdf]

Full gel scan manuscript

pJNK

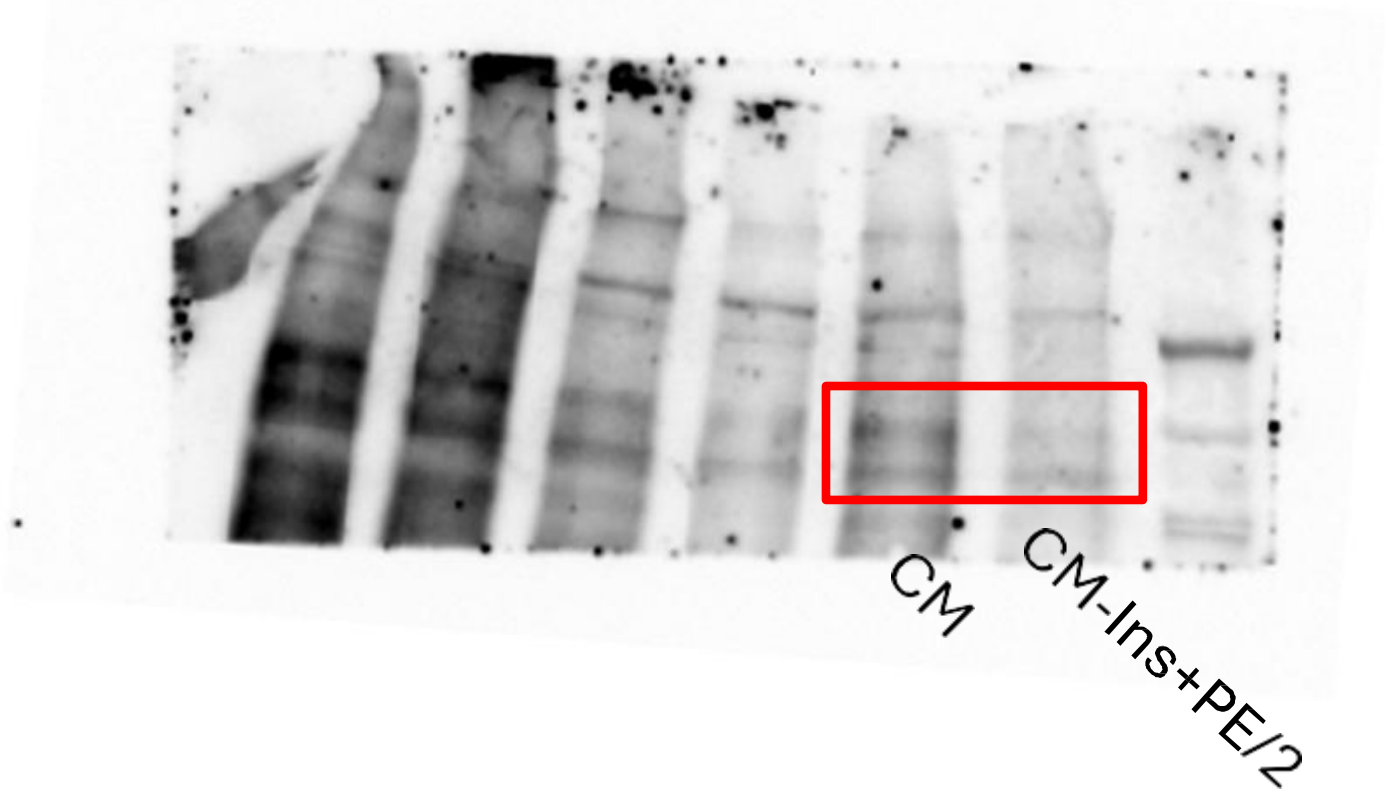

JNK

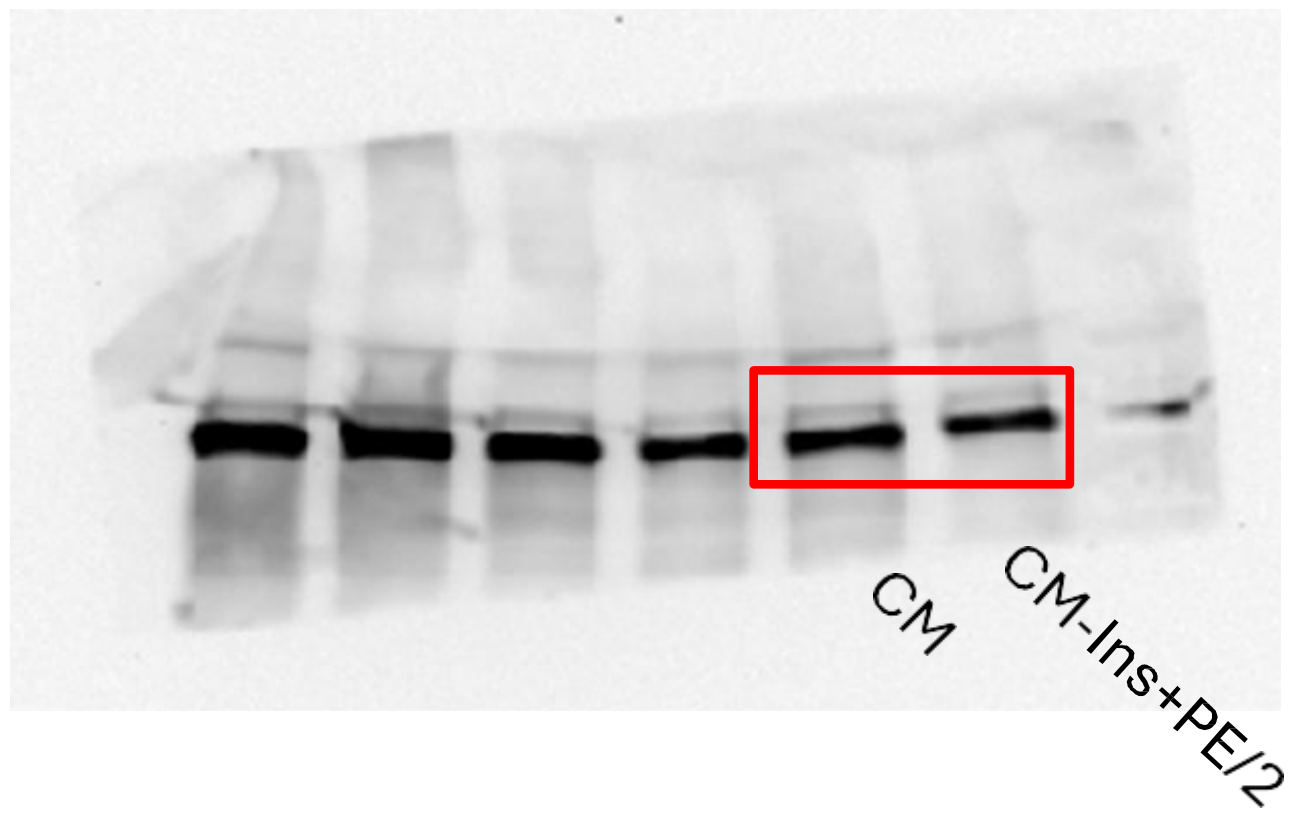

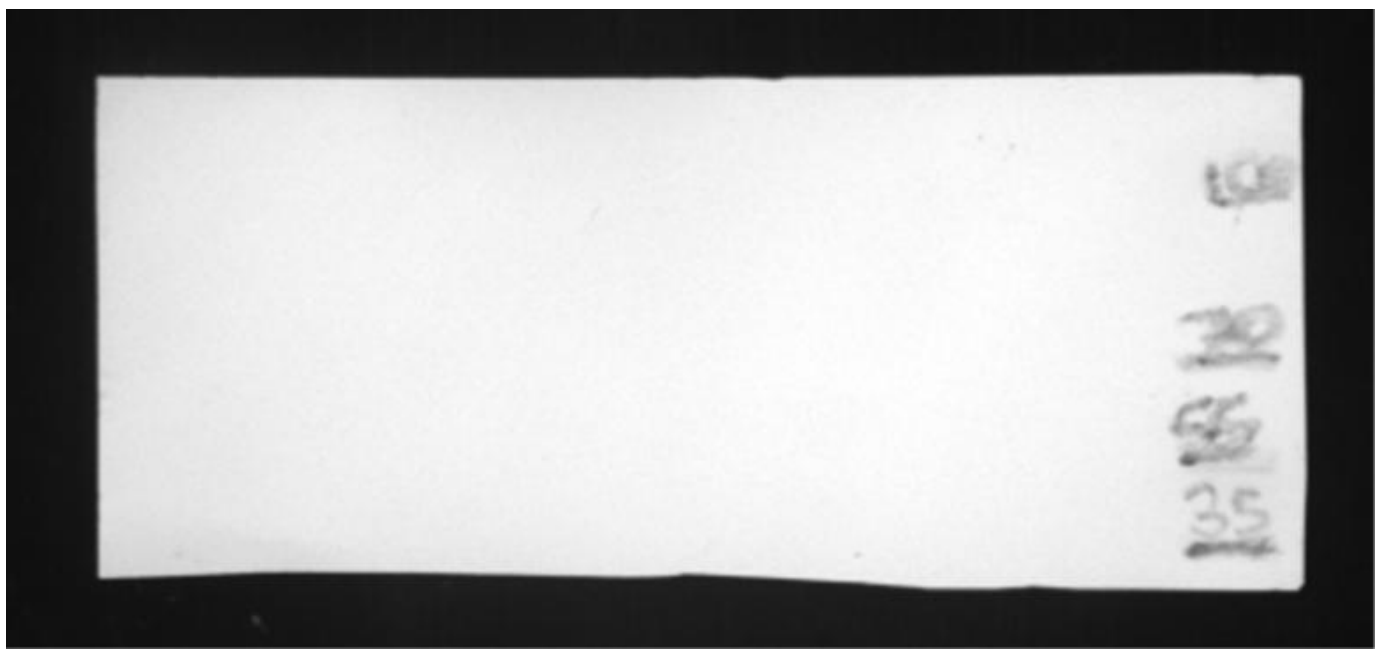

VINCULIN

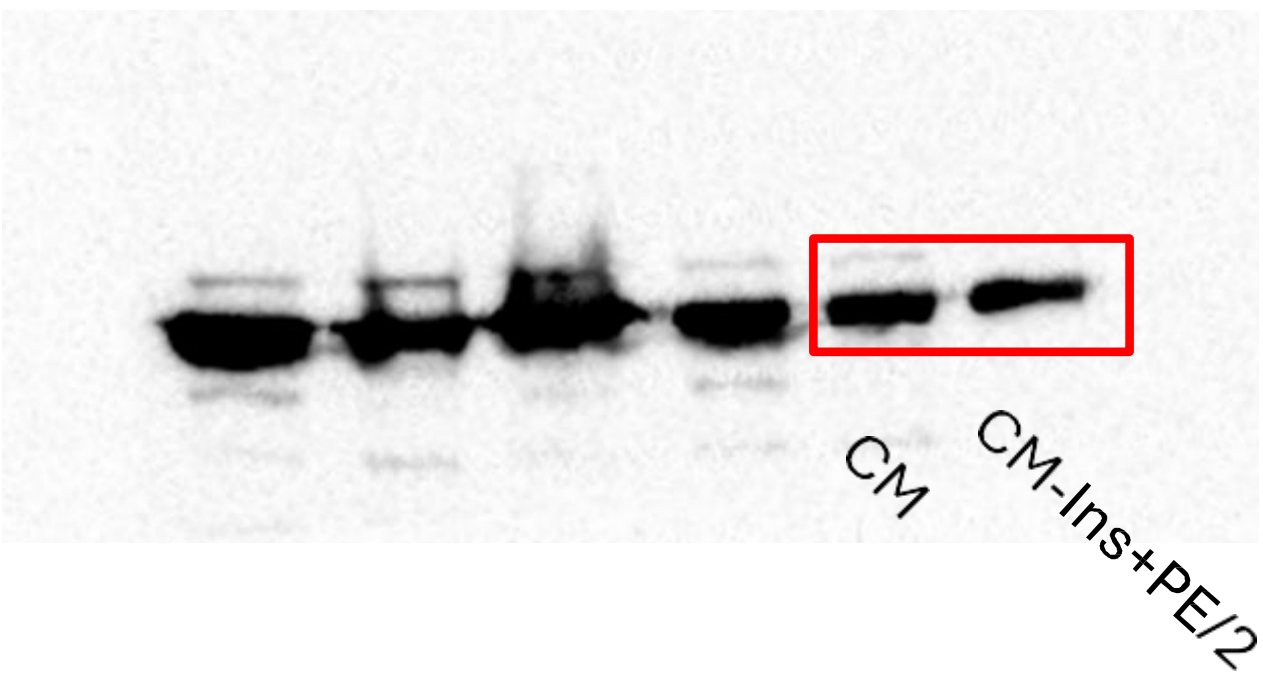

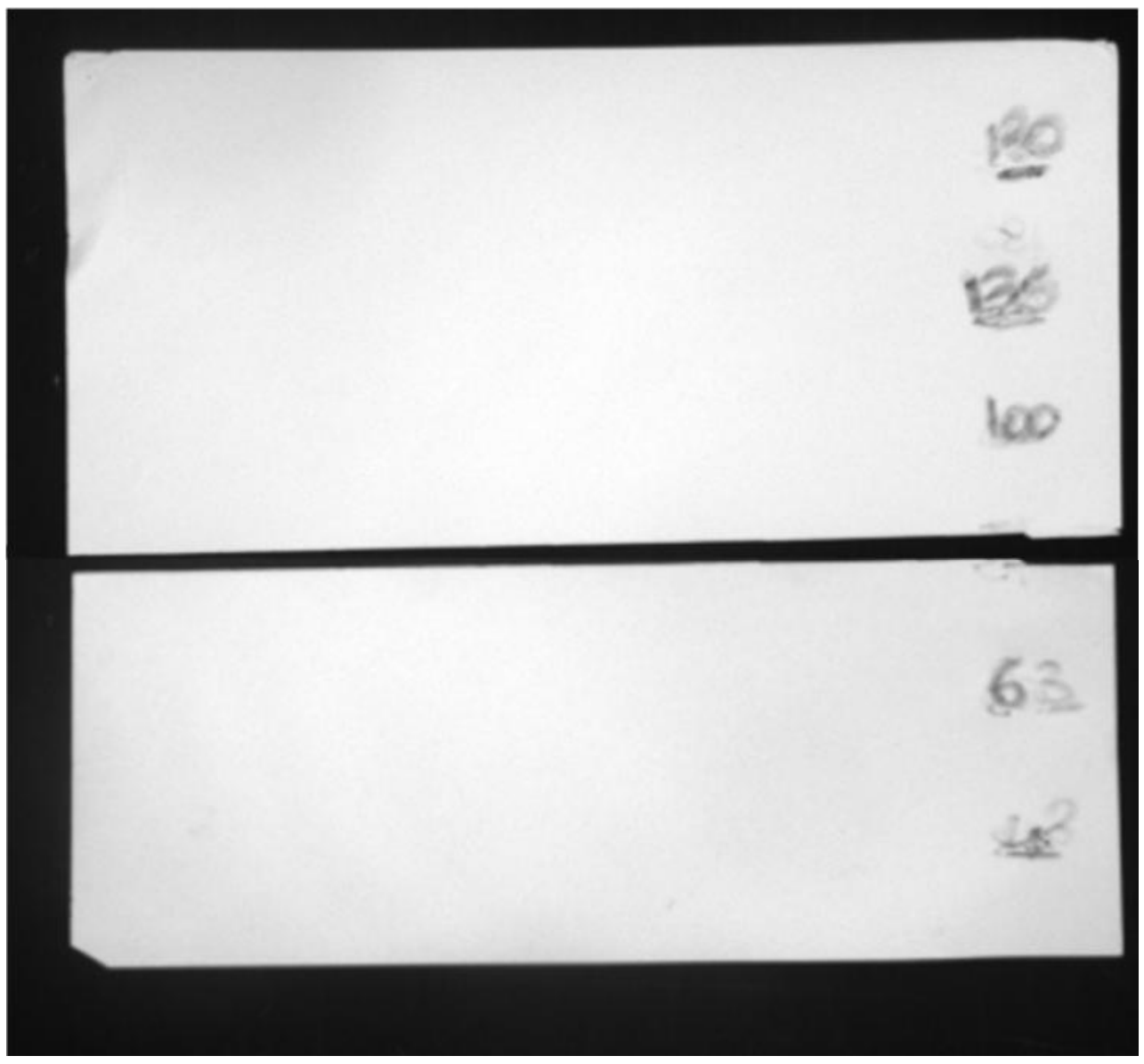

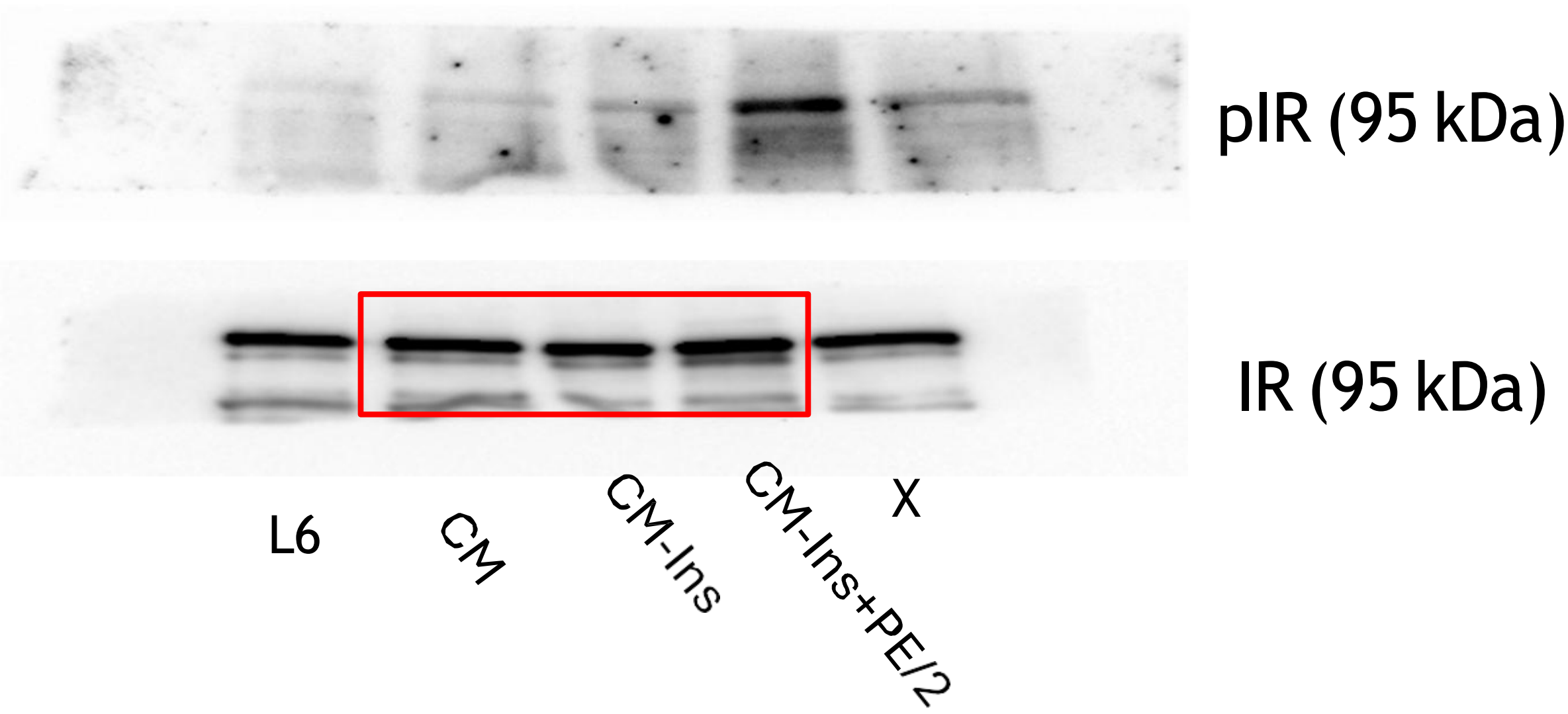
pIR (95 kDa)
IR (95 kDa)
L6
CM
CM-Ins
CM-Ins+PE/2
X

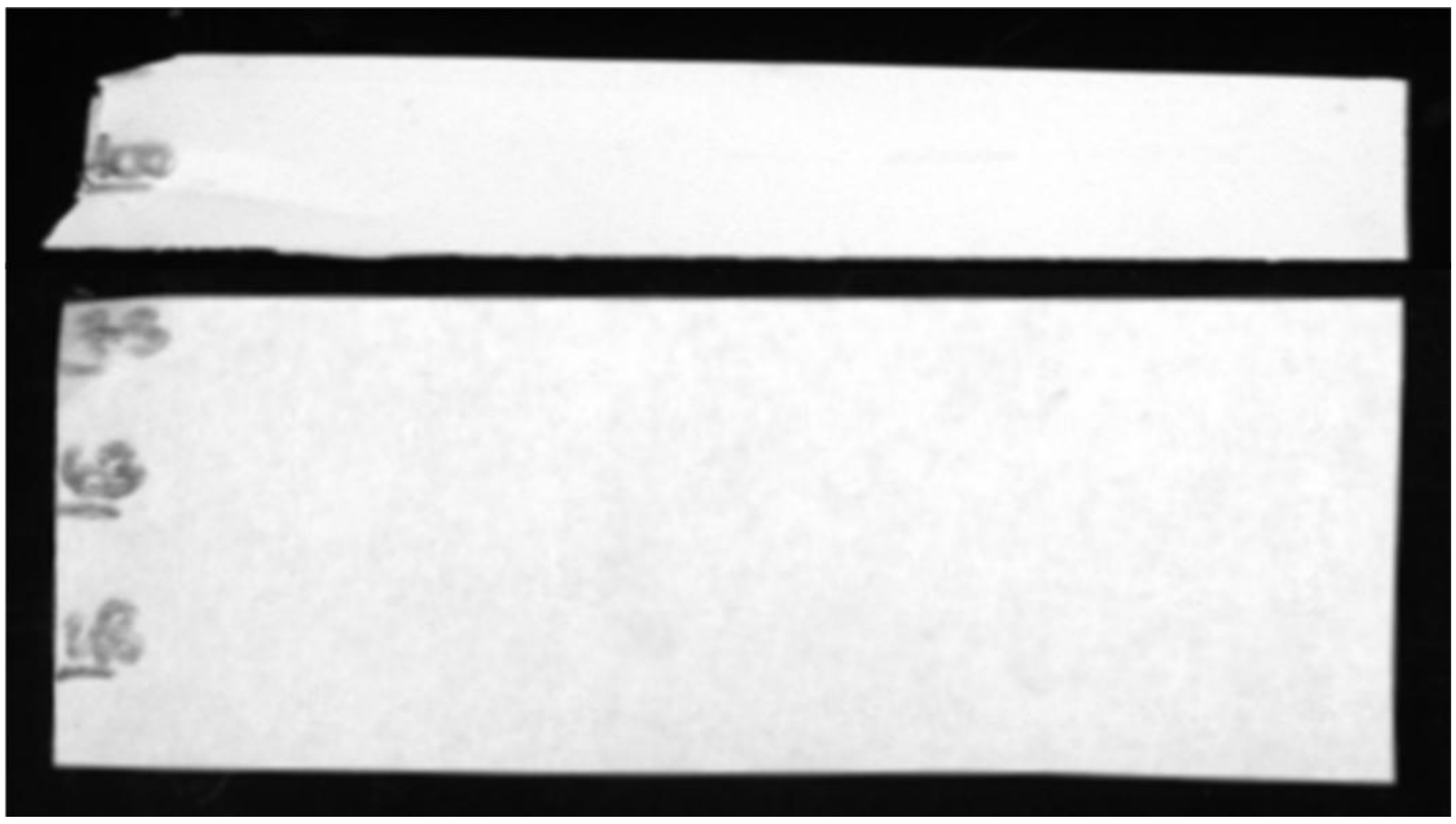

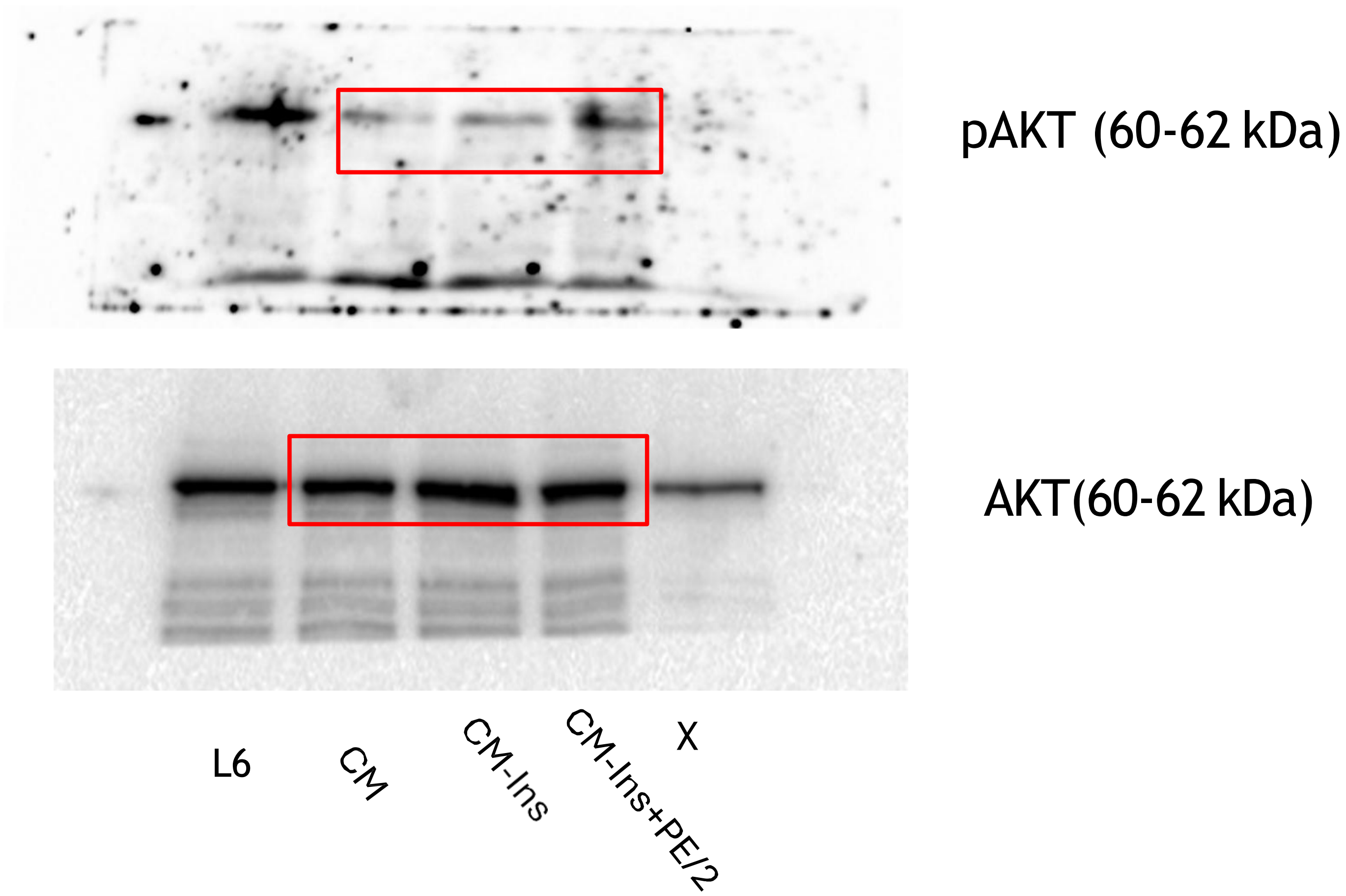
pAKT (60-62 kDa)
AKT(60-62 kDa)
L6
CM
CM-Ins
CM-Ins+PE/2
X

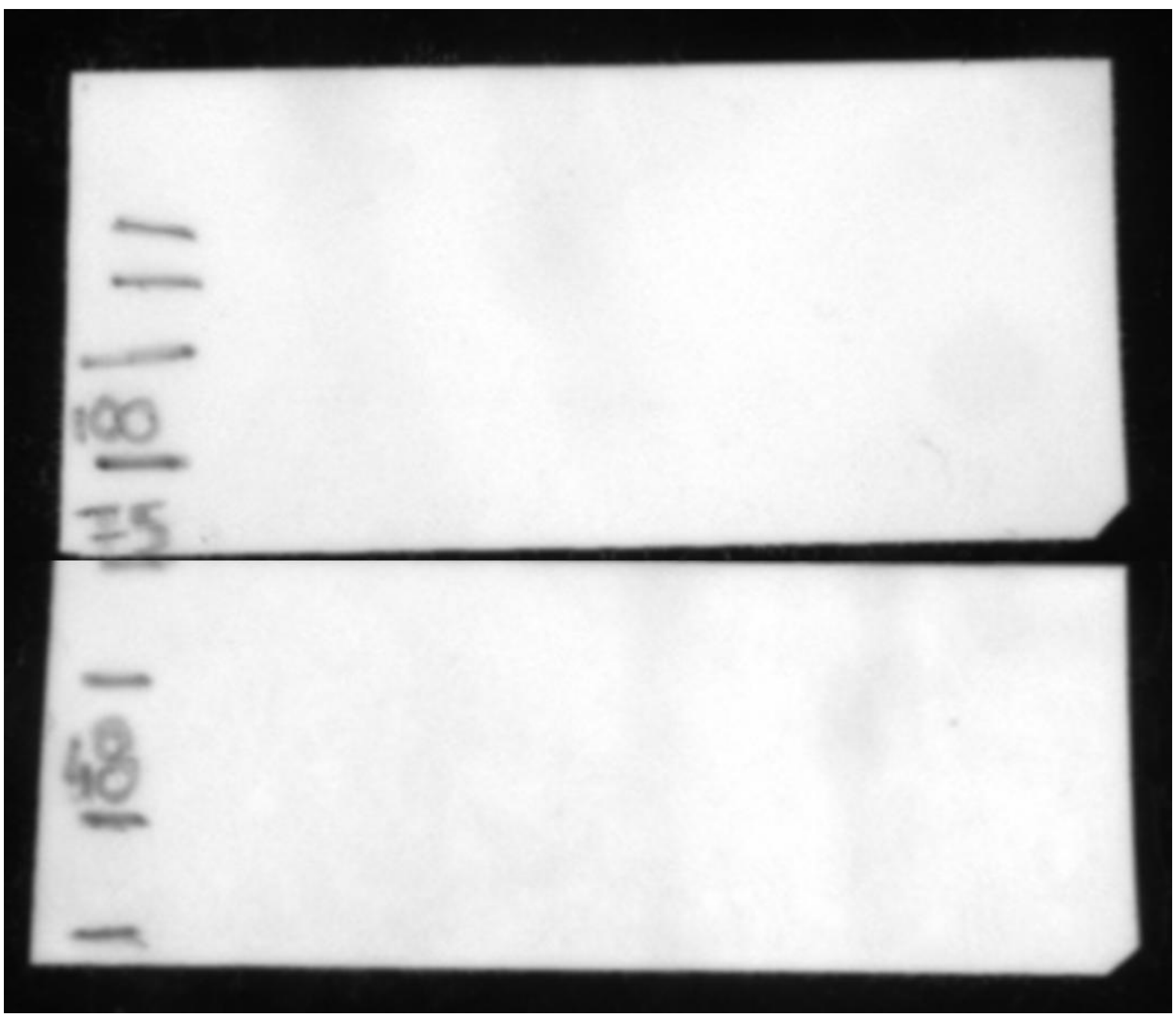

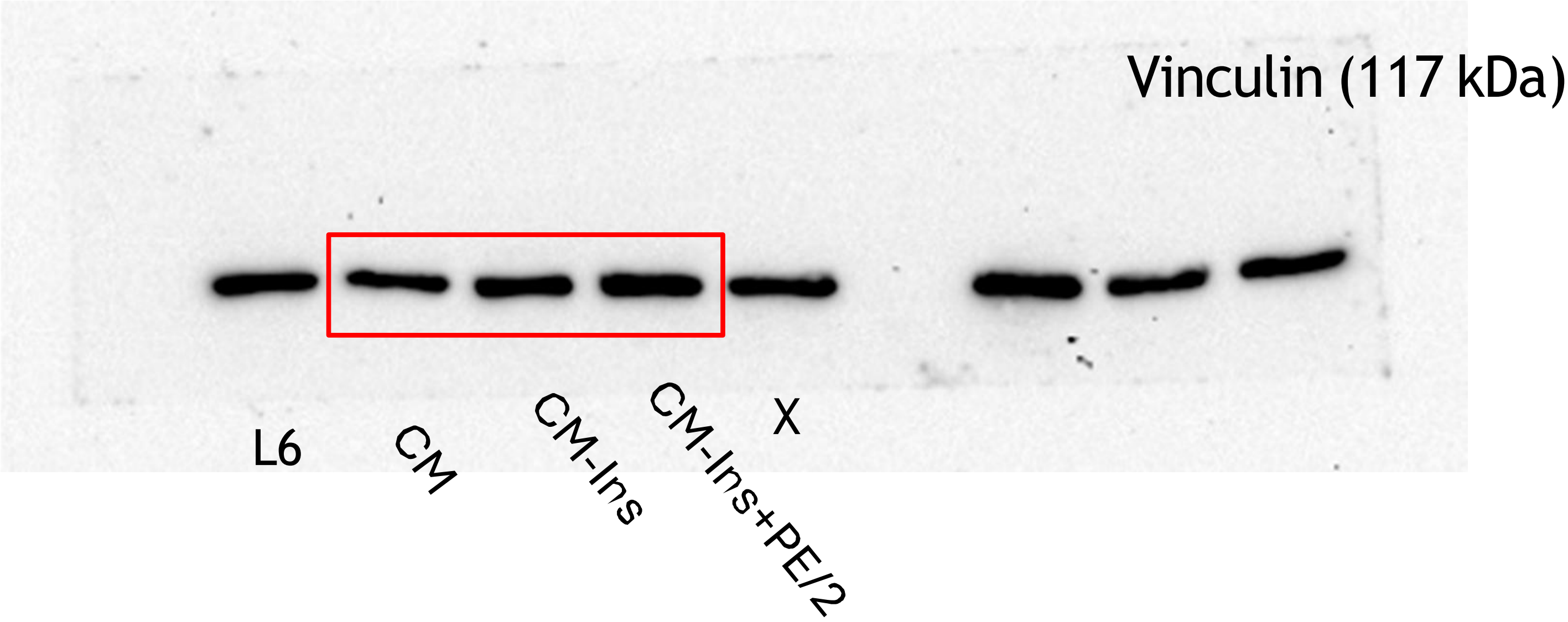
Vinculin (117 kDa)
L6
CM
CM-Ins
CM-Ins+PE/2
X

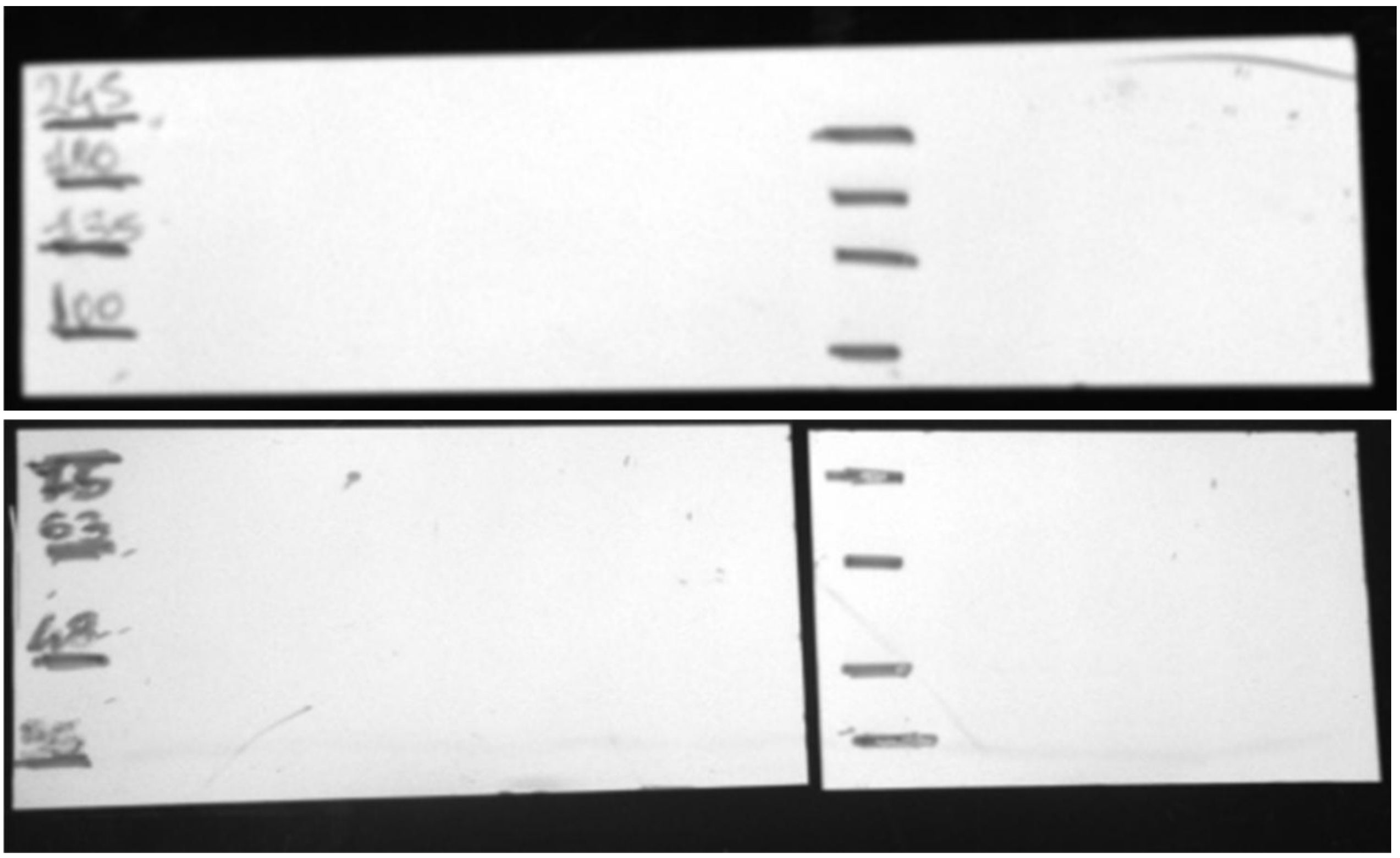
245
100
63
48

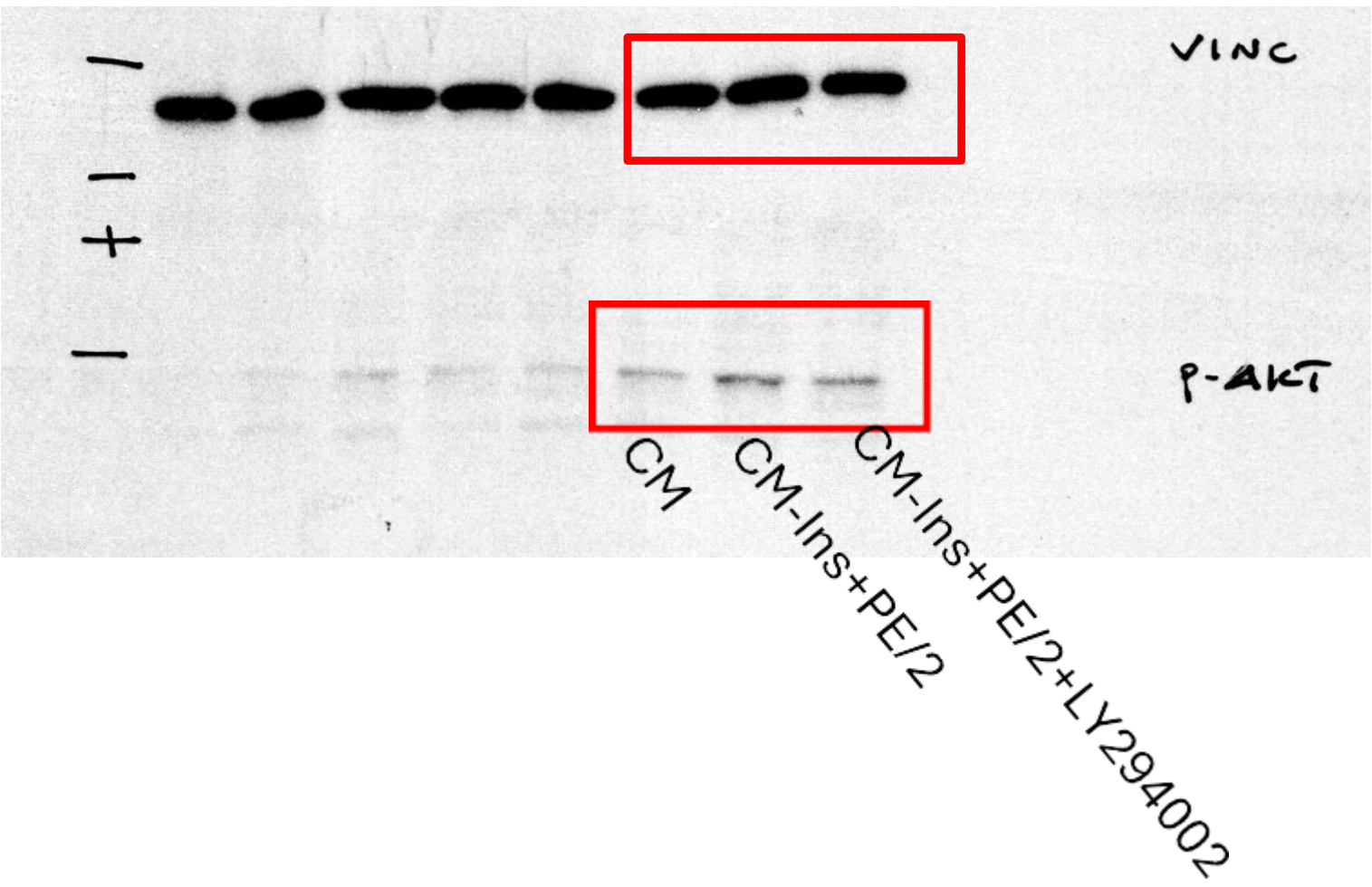

Vinculin (117 kDa)

pAKT (60-62 kDa)

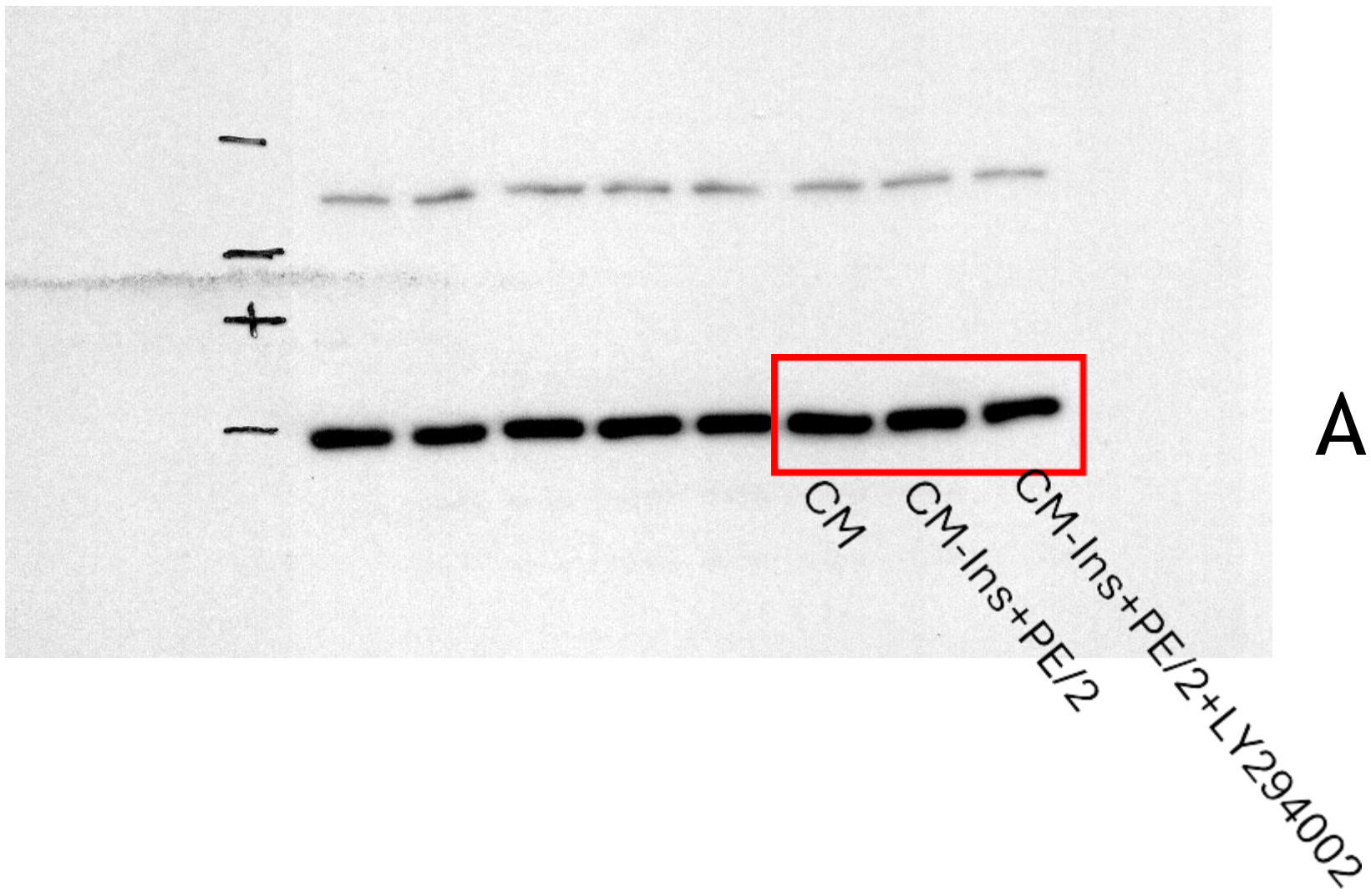

AKT (60-62 kDa)

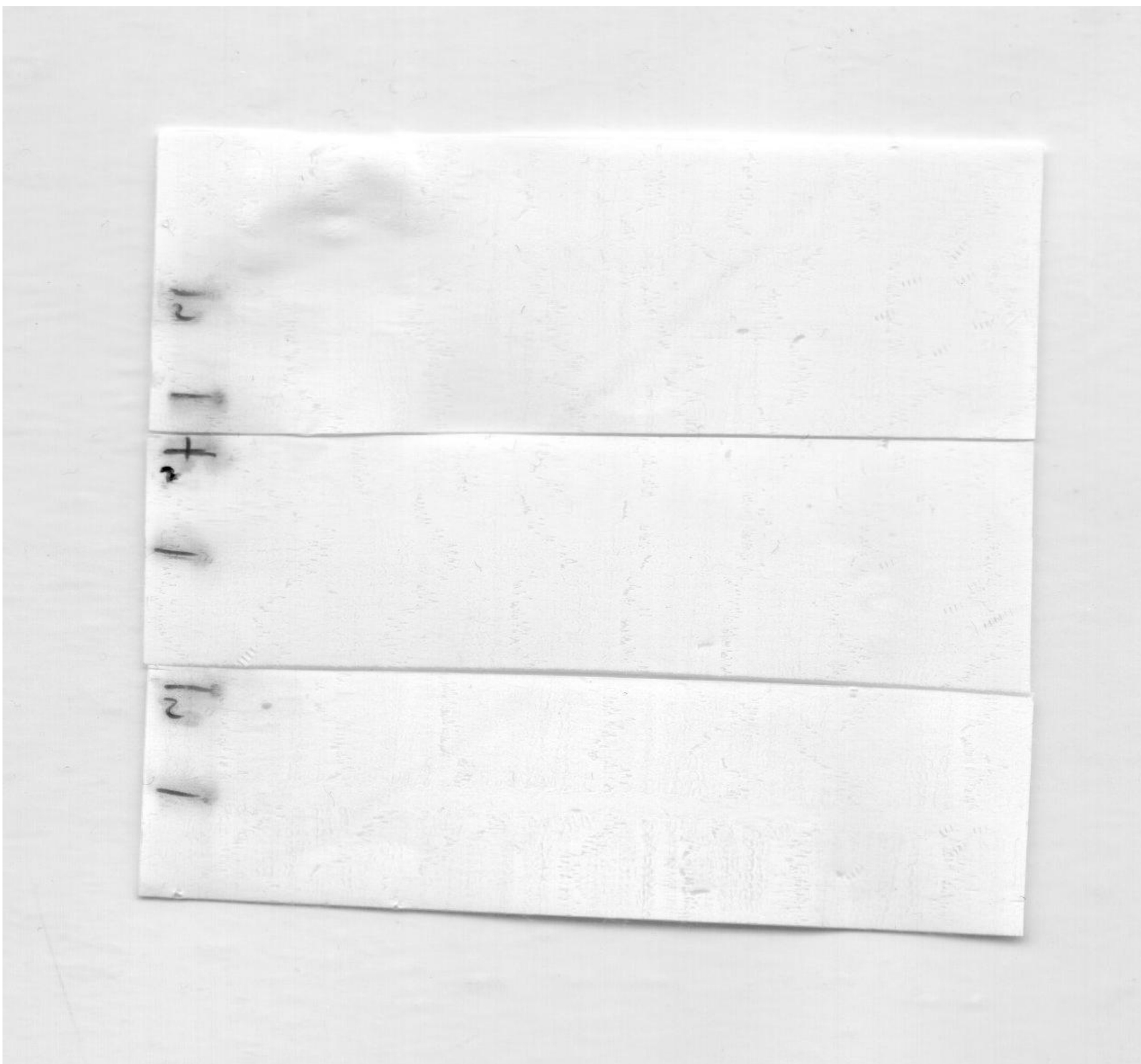

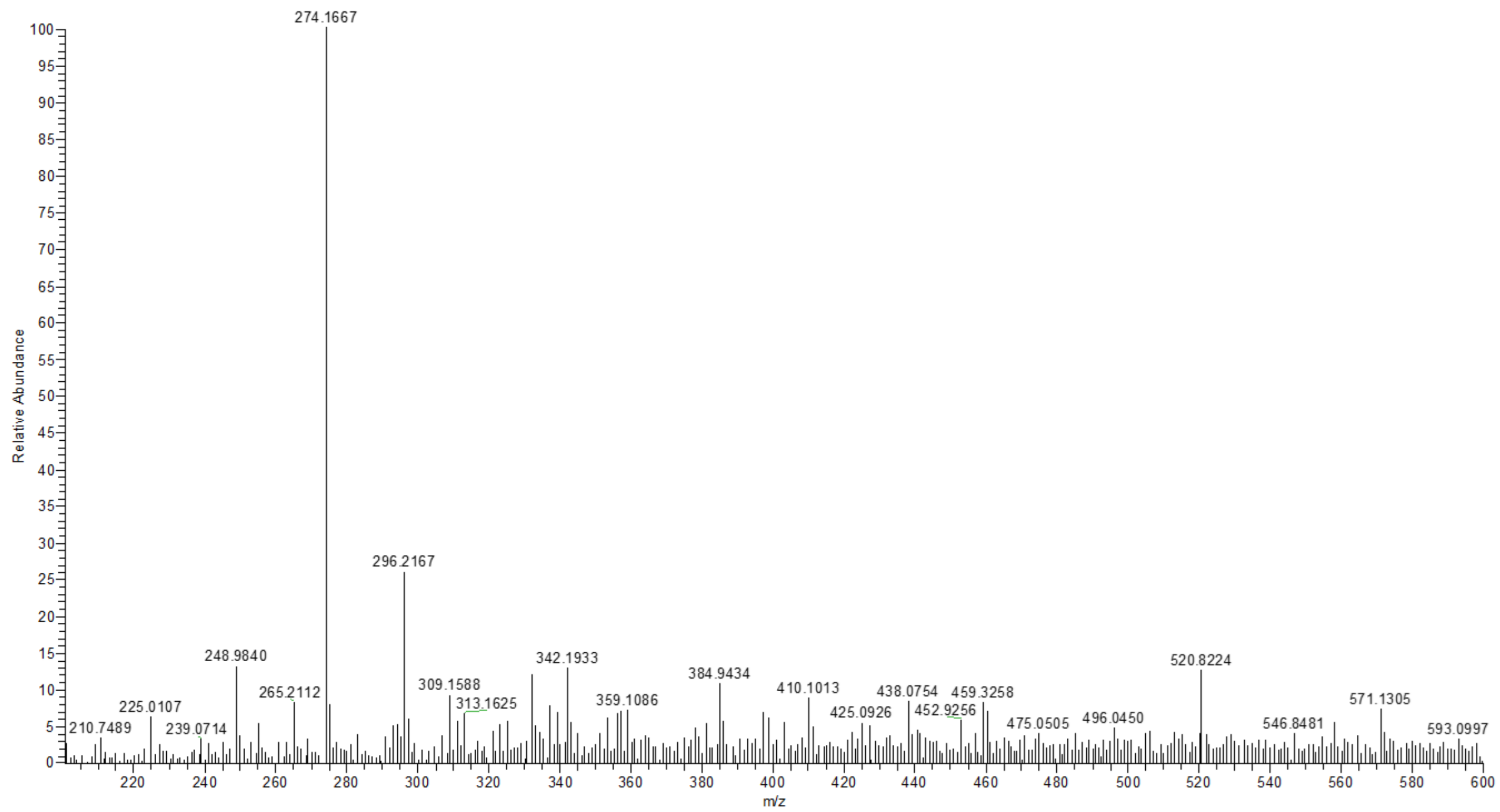

HR-ESI-MS spectrum of compound PE/2 acquired in negative-ion mode
