# Supplementary material for: Conditioned media derived from HepG2 cells treated with phosphoeleganin synthetic-derived compound PE/2 improve cytokine balance and metabolic response in 3T3-L1 adipocytes
Source: Front Pharmacol. 2026 Oct 23;17:1909848. doi: 10.3389/fphar.2026.1909848 (PMC13739515; doi:10.3389/fphar.2026.1909848)
Supplement: Supplementary file 2 [file Table1.docx]

| **PRIMERS** | **SEQUENCES** |
| --- | --- |
| **Cyclophilin A** | Forward: 5'-GCAGACAAAGTTCCAAAGACAG-3'  Reverse: 5'-CACCCTGGCACATGAATCC-3' |
| **TNFα** | Forward 5'-AGCCCCCAGTCTGTATCCTT-3'  Reverse 5'-CTCCCTTTGCAGAACTCAGG-3' |
| **IL6** | Forward: 5'-GCCAGAGTCCTTCAGAGAGA-3'  Reverse: 5'-GGTCTTGGTCCTTAGCCACT-3' |
| **IL1β** | Forward: 5'-AACCTTTGACCTGGGCTGTC-3'  Reverse: 5'-CCTCATCCTGGAAGGTCCAC-3' |
| **IL8** | Forward: 5'-AGCCACCCGCTCGCTTCTCT-3'  Reverse: 5'-TCGGTTTGGGTGCAGTGGGG-3' |
| **SREBP1c** | Forward 5'-GCCCCTGCCCACCTCAAACC-3'  Reverse 5'-GCACTGGCACGGGCATCCTT-3' |
| **ChREBP** | Forward 5'-GTCCGATATCTCCGACACACTCTT-3'  Reverse 5'-CATTGCCAACATAAGCATCTTCTG-3' |
| **FAS** | Forward 5'-ACGACAGCAACCTCACGGCG-3'  Reverse 5'-CTGCAGAGCCCGTGCCAGAC-3' |
| **C/EBPα** | Forward 5’-TGGACAAGAACAGCAACG-3'  Reverse 5’-GTCAACTCCAGCACCTTC-3' |
| **Adiponectin** | Forward 5'-GACGACACCAAAAGGGCTCA-3'  Reverse 5'-GAGTGCCATCTCTGCCATCA-3' |
| **GLUT4** | Forward 5'-TGCTCTCCGGTTCCGTGGGT-3'  Reverse 5'-GGTTCCCCATCGTCAGAGCCG-3' |
| **FABP4** | Forward 5'-AATCACCGCAGACGACAG-3'  Reverse 5'-ACGCCTTTCATAACACATTCC-3' |
| **PPARγ** | Forward: 5'-CAGTGGAGACCGCCCAGGCT-3' Reverse: 5'-TGGAGCAGGGGGTGAAGGCT-3' |

**Primer sequences used in Real-time RT-PCR analysis.**
